# Integrated functional genomic analysis identifies regulatory variants underlying a major QTL for disease resistance in European sea bass

**DOI:** 10.1186/s12915-025-02180-4

**Published:** 2025-03-11

**Authors:** Robert Mukiibi, Serena Ferraresso, Rafaella Franch, Luca Peruzza, Giulia Dalla Rovere, Massimiliano Babbucci, Daniela Bertotto, Anna Toffan, Francesco Pascoli, Sara Faggion, Carolina Peñaloza, Costas S. Tsigenopoulos, Ross D. Houston, Luca Bargelloni, Diego Robledo

**Affiliations:** 1 The Roslin Institute and Royal (Dick), University of Edinburgh, Edinburgh, EH25 9RG UK; 2 Department of Comparative Biomedicine and Food Science, University of Padova, Legnaro, 35020 Italy; 3 Istituto Zooprofilattico Sperimentale delle Venezie, National Reference Laboratory for Fish Diseases, Legnaro, 35020 Italy; 4 Benchmark Genetics, Roslin Innovation Centre, Edinburgh, EH25 9RG UK; 5 Institute of Marine Biology, Biotechnology and Aquaculture (IMBBC), Hellenic Centre for Marine Research (HCMR), Heraklion, 715 00 Greece; 6 Department of Zoology, Genetics and Physical Anthropology, Universidade de Santiago de Compostela, Santiago de Compostela, 15706 Spain

**Keywords:** GWAS, eQTL, Multi-omics, SNP, *Dicentrarchus labrax*, Viral nervous necrosis, Disease resistance, Aquaculture, Interferon

## Abstract

**Background:**

Viral nervous necrosis (VNN) is an important viral disease threatening global aquaculture sustainability and affecting over 50 farmed and ecologically important fish species. A major QTL for resistance to VNN has been previously detected in European sea bass, but the underlying causal gene(s) and mutation(s) remain unknown. To identify the mechanisms and genetic factors underpinning resistance to VNN in European sea bass, we employed integrative analyses of multiple functional genomics assays in European sea bass.

**Results:**

The estimated heritability of VNN resistance was high (*h*^2^ ~ 0.40), and a major QTL explaining up to 38% of the genetic variance of the trait was confirmed on chromosome 3, with individuals with the resistant QTL genotype showing a 90% survivability against a VNN outbreak. Whole-genome resequencing analyses narrowed the location of this QTL to a small region containing 4 copies of interferon alpha inducible protein 27-like 2A (*IFI27L2A*) genes, and one copy of the interferon alpha inducible protein 27-like 2 (*IFI27L2*) gene. RNA sequencing revealed a clear association between the QTL genotype and the expression of two of the *IFI27L2A* genes, and the *IFI27L2* gene. Integration with chromatin accessibility and histone modification data pinpointed two SNPs in active regulatory regions of two of these genes (*IFI27L2A* and *IFI27L2*), and transcription factor binding site gains for the resistant alleles were predicted. These alleles, particularly the SNP variant CHR3:10,077,301, exhibited higher frequencies (0.55 to 0.77) in Eastern Mediterranean Sea bass populations, which show considerably higher levels of resistance to VNN, as compared to susceptible West Mediterranean and Atlantic populations (0.15–0.25).

**Conclusions:**

The SNP variant CHR3:10,077,301, through modulation of *IFI27L2* and *IFI27L2A* genes, is likely the causative mutation underlying resistance to VNN in European sea bass. This is one of the first causative mutations discovered for disease resistance traits in fish and paves the way for marker-assisted selection as well as biotechnological approaches to enhance resistance to VNN in European sea bass and other susceptible species.

**Supplementary Information:**

The online version contains supplementary material available at 10.1186/s12915-025-02180-4.

## Background

The European sea bass (*Dicentrarchus labrax*) is a highly esteemed marine fish in Europe and the Mediterranean, boasting major economic and cultural value [1]. Over the past two decades, global aquaculture production of European sea bass has experienced remarkable growth, steadily increasing from 7694 tons in 2000 to 299,810 tons in 2021; the major producing countries are Turkey, contributing approximately 52%, and Greece, accounting for around 17% of the total global production [2]. Aquaculture dominates European sea bass production, constituting 98% of the total, as wild fisheries production has witnessed a steady decline of 38% over the past decade [2]. However, the industry faces an increasing challenge in the form of infectious diseases, which pose a significant threat to both the sustainability of the industry and the welfare of farmed European sea bass. Infectious diseases currently account for approximately 10% of all fish mortalities on aquaculture farms [3].

Viral nervous necrosis (VNN) stands as the main viral infectious disease, currently responsible for 15% of total on-farm infectious disease-related mortalities in European sea bass [3]. Beyond the direct toll of mortalities, sea bass producers also suffer economic losses stemming from fish that survive the viral infection but experience delayed growth [4]. Primarily affecting larvae and juvenile fish, VNN inflicts moderate to high mortality rates, reaching up to 100% in affected farms [5]. The causative agent of VNN, nervous necrosis virus (NNV), belongs to the *Betanodavirus* genus in the *Nodaviridae* family, and is a non-enveloped single positive-stranded RNA virus [5]. The main target of NNV is the central nervous system, particularly the brain, spinal cord and the retina, where viral replication and multiplication occurs [6]. NNV has a broad host range, as it can infect a wide range of aquaculture fish species such as Asian seabass (*Lates calcarifer*), greasy grouper (*Epinephelus tauvina*), red-spotted grouper (*Epinephelus akaara*), gilthead sea bream (*Sparus aurata*), and turbot (*Scophthalmus maximus*) [6–8]. Effective prevention and treatment strategies for VNN remain elusive, and therefore current practices focus on minimizing disease outbreaks through stringent biosecurity measures, vaccination, and developing experimental therapeutics [6, 7].

Recent studies have revealed significant phenotypic and genetic variation in resistance to VNN within farmed European sea bass populations. Moderate to high heritability estimates of 0.10 to 0.43 have been reported for this trait [9–13], indicating the potential for selective breeding to develop strains with heightened genetic resistance to the virus. Notably, a major quantitative trait locus (QTL) with a large impact on VNN resistance has been identified on chromosome 3 (previously LG22) of the sea bass genome in three distinct farmed populations [9, 11, 12, 14]. This major QTL accounts for over 33% of the genetic variation in resistance to VNN [9, 11, 12, 14], thus marker-assisted selection can drive rapid and significant improvement in this trait, leading to sustained reductions in disease outbreaks.

Moreover, understanding the genomic basis underlying this QTL would open avenues for leveraging genome engineering approaches to bolster VNN resistance in populations and species lacking genetic variation at this resistance locus. Several genes, including *PLK4*,* HSPA4L* and* REEP1*, have been suggested as potential genes underlying VNN resistance due to their proximity to the QTL and their recognized antiviral function [12]. In a recent large study by Delpuech et al. on a large population, the putative causative mutation was proposed to reside approximately 1.9 and 6 kb downstream of the *ZDHHC14* and IFI27-like genes on chromosome 3, the same chromosome/linkage group where previous QTLs for the same trait were reported, albeit at a considerable distance [14]. In the same study, other less prominent QTLs were identified on the same chromosome 3. It is worth noting that all these studies were conducted on a highly fragmented genome assembly [15], and so far no functional evidence in support of any causative gene has been reported.

The availability of highly contiguous and well-annotated genomes is essential to uncover the causative variants underlying QTL [16]. In recent years, new high-quality genome assemblies have been released for many aquaculture species [17–19], including the European sea bass (GCA_905237075.1, contig N50 = 12 Mb). However, the functional annotation of these assemblies is mostly limited to gene positions along the genome, facilitated by the abundant collection of RNA sequencing datasets for many teleost species and the general conservation of coding sequences. Considering the key role of regulatory variants in shaping the genetic architecture of complex traits [20, 21], it is crucial to extend the functional annotation to the non-coding regions of the genome to advance toward the identification of causative variants underlying QTLs.

Recent studies, such as large genotype-tissue expression analyses in humans [22], cattle [23] and pigs [24], have identified expression QTLs (eQTLs) across different tissues. Notably, some of these eQTLs co-localize with QTL regions strongly associated with complex traits of both health and economic or production significance. This evidence underscores the importance of identifying loci modulating gene expression (eQTLs) and colocalizing with disease resistance QTLs, which can significantly contribute to unraveling the genetic architecture of complex traits, including disease resistance to pathogenic infections. Additionally, novel tools to identify and profile the regulatory regions of the genomes have become available in recent years, such as the assay for transposase-accessible chromatin sequencing (ATAC-seq) [25], and the chromatin immunoprecipitation sequencing (ChIP-seq) [26]. Integrating multiple omics evidence with QTL/eQTL data is pivotal for the reliable identification of causal genetic variants underlying variability in a complex trait; however, these datasets are scarce non-model species.

In this study, we investigated the genetic architecture and underlying functional genomic basis of resistance to VNN in a non-model species, the European sea bass. By combining whole-genome genotyping, targeted eQTL identification, analysis of chromatin accessibility and ChIP-seq data, and population genomic evidence, we unveiled reliably the putative mechanisms driving a major QTL for resistance to VNN in this species. This integrative approach provides a comprehensive understanding of the genetic factors contributing to disease resistance and sheds light on potential avenues for future research and breeding strategies in non-model organisms like the European sea bass.

## Results

### VNN infection challenge, survival analysis, and population genetic structure

**Fig. 1:**
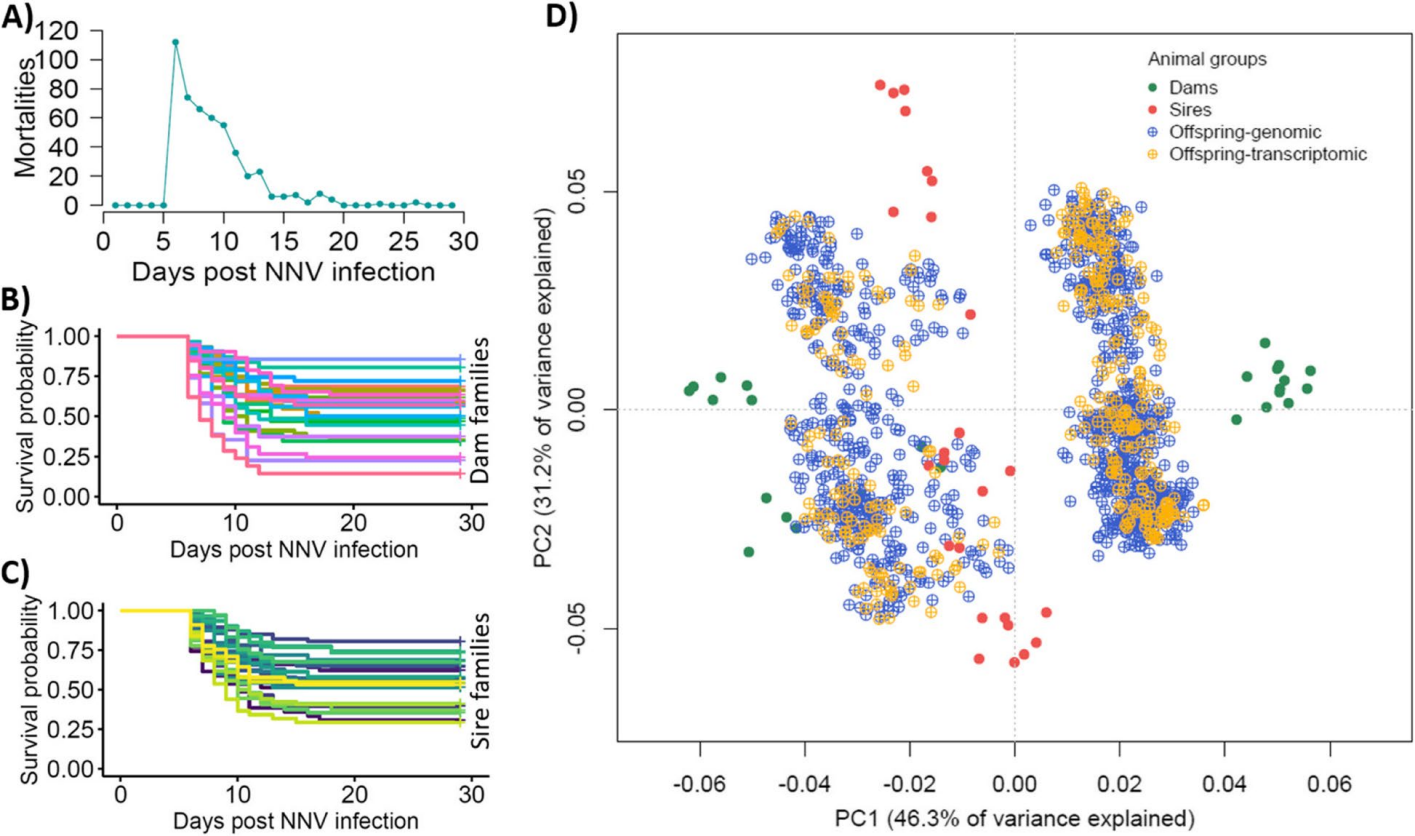
VNN challenge experiment results and genetic structure of the European sea bass challenged population.** A** Number of mortalities each day of the challenge experiment. **B** Survival curves for each dam family throughout the experiment. **C** Survival curves for each sire family throughout the experiment. **D** Principal component analyses (based on SNP array genotypes) showing the genetic structure of the study population

No fish mortality was observed during the first 5 days of the VNN challenge experiment; however, the peak of mortality was observed on day 6, and then the number of deaths decreased steadily reaching zero on day 29, when the experiment was terminated (Fig. 1A). The overall mortality of the challenge experiment was 46.84% (Fig. 1A), close to that observed in previous studies with similar experimental design [10, 27].

All challenged animals and their parents were genotyped using a 30 K SNP array, and then imputed to whole-genome genotypes from 90 whole-genome sequenced individuals (all 50 parents and 40 offspring). Genotypes were used to reconstruct the pedigree. Each sire and dam contributed a substantial number of offspring to the challenge population (Additional file 1: Figure S1), and a survival analysis showed phenotypic variation in resistance to VNN infection between both dam and sire families, with overall survival ranges of 14.3–85.7% for dam families (Fig. 1B), and 29.2–80.6% for sire families (Fig. 1C).

Principal component analysis of parents and offspring showed important genetic structure, likely driven by the geographic origin of the parents, especially that of the dams (Fig. 1D). In fact, the base population for the breeding programme at the hatchery that provided the biological material for the present study was established by crossing European sea bass of Atlantic and Mediterranean origin populations, which have been consistently reported to show significant genetic divergence [15].

### Genetic parameters and association study

Heritability estimates for VNN resistance were moderate to high, ranging between 0.35 and 0.45 (Table 1). These estimates are in general higher than those reported in previous studies using different farmed sea bass populations [9–12, 14]. Heritabilities were slightly higher when calculated using whole-genome genotypes, and the heritability of days to death (DD) was higher than for binary survival (BS) (Table 1). There was high genetic correlation between BS and DD (0.99, Table 1); therefore, both measurements represent exactly the same trait.

**Table 1:** Heritabilities and genetic correlation for resistance to VNN; days to death (DD) and binary survival (BS)

	***h***^**2**^** ± se (BS)**	***h***^**2**^** ± se (DD)**	***r***_***g***_** ± se (BS & DD)**
SNP array	0.35 ± 0.05	0.39 ± 0.05	0.99 ± 0.00
Imputed WGS	0.40 ± 0.06	0.45 ± 0.06	-
Pedigree-based	0.38 ± 0.10	0.23 ± 0.08	-

**Fig. 2:**
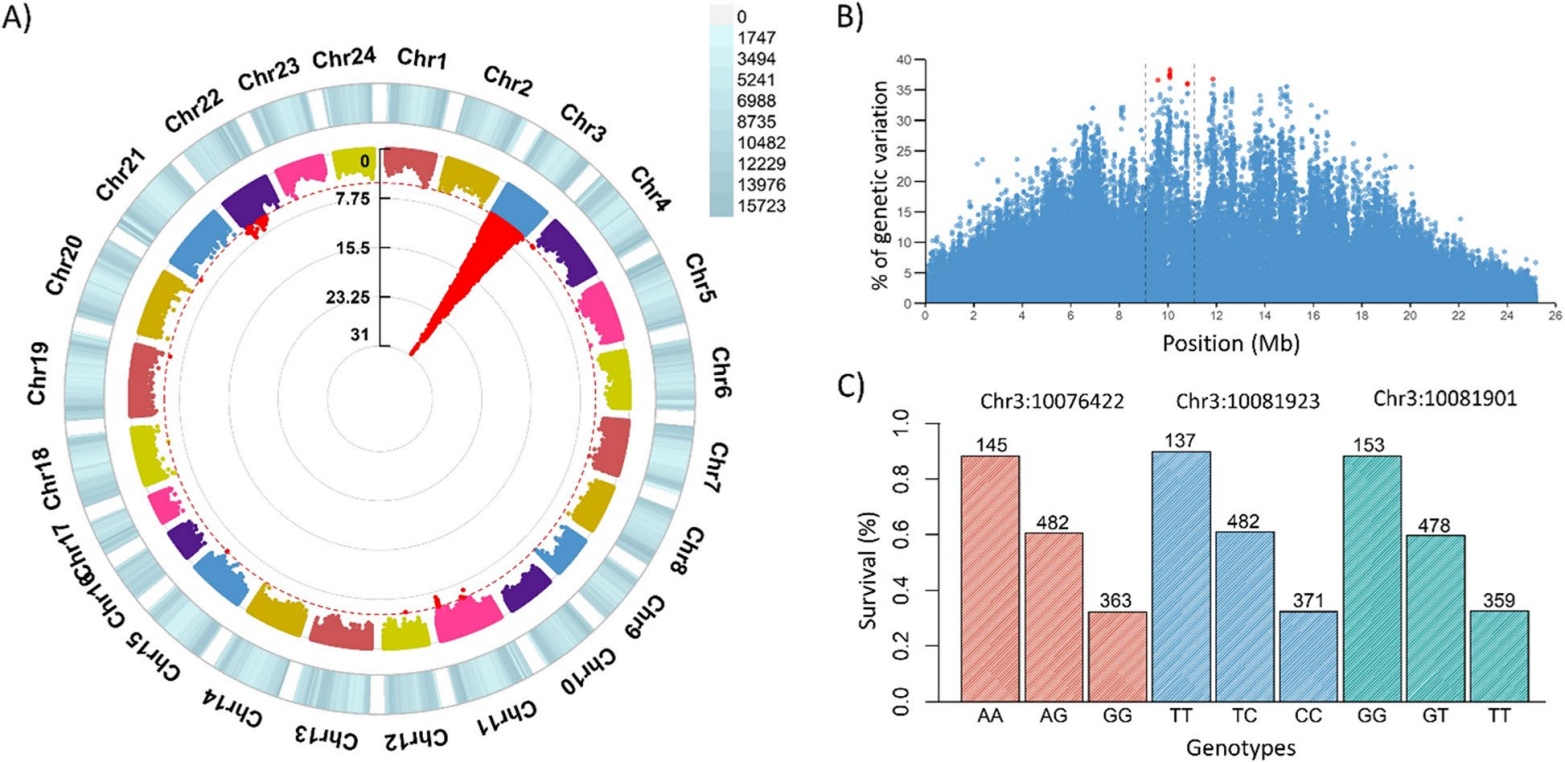
Signatures of resistance to VNN in the European sea bass genome.** A** Circular Manhattan plot showing associations results between WGS variants and VNN resistance (dotted red line indicates genome-wide significance threshold of FDR < 0.05). **B** Percentage of genetic variance explained by the variants on Chromosome 3. **C** Barplot showing the percentage of survival of the animals with each genotype for the three most significant SNPs

GWAS analysis for resistance to VNN revealed a major QTL in chromosome 3 (Fig. 2A). The significant markers explained up to 15.27% of the phenotypic variance, and up to 38.28% of the genetic variance in survival to VNN (Fig. 2B and Additional file 2: Table S1 & Additional file 2: Table S2). The use of whole-genome sequencing improved the power of these analyses, as the variants explaining the largest percentage of genetic variance were not present in the SNP array [28]. Consistently with the high percentage of variance explained, the QTL genotype had a large and additive impact on survival rate, as fish homozygous for the resistance genotype showed 88.2–89.8% survival, heterozygous individuals 59.6–61.0%, and homozygous animals for the susceptible genotype 32.2–32.6% (Fig. 2C). Thus, implying that the homozygous-resistant fish showed ~ 55% increased survival compared to homozygous susceptible fish.

### Annotation of the QTL region

**Fig. 3:**
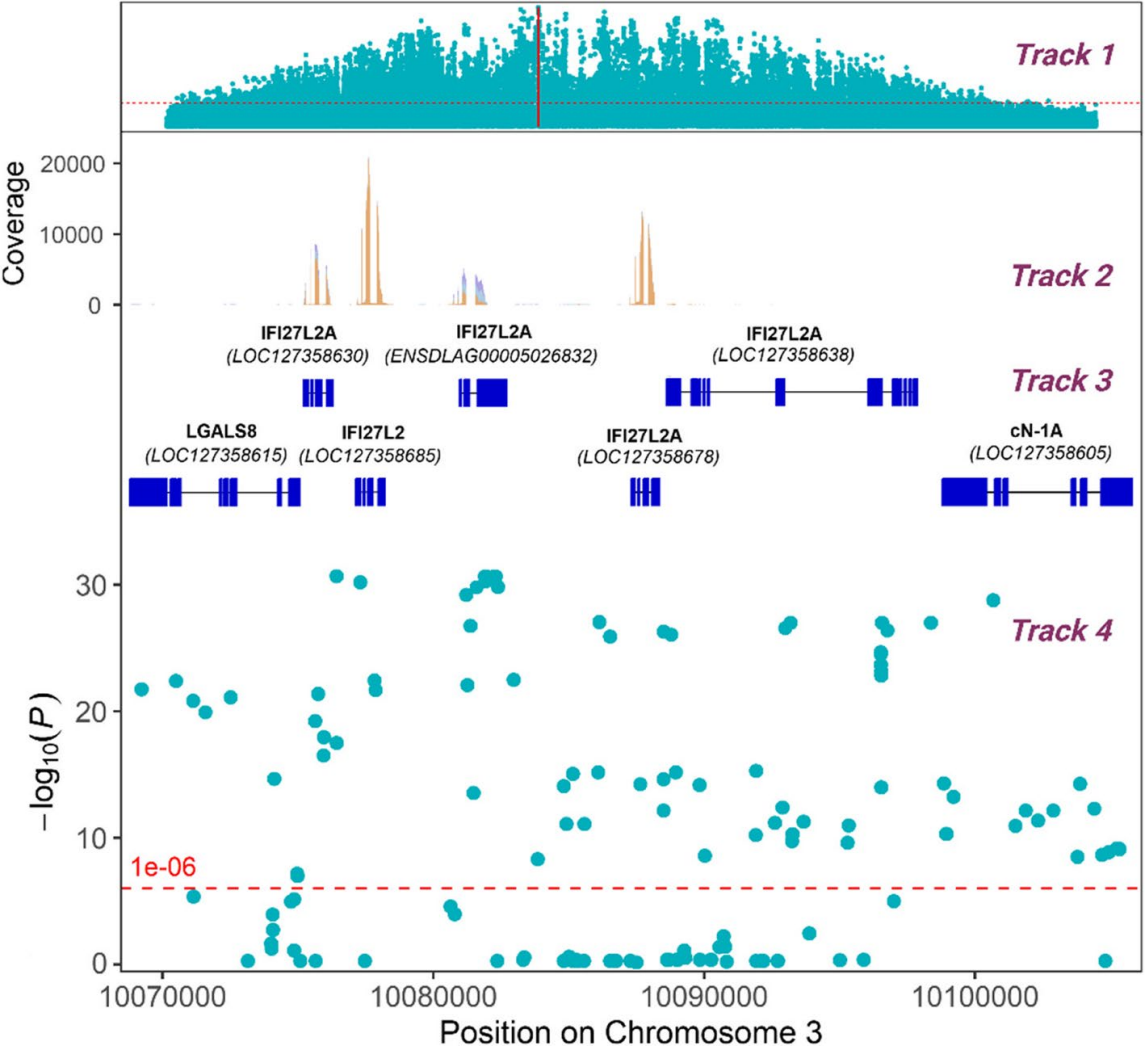
Functional annotation of the VNN resistance major QTL region in European sea bass chromosome 3. The region containing the variants showing the highest association with VNN resistance (Chromosome 3: 10,068,810–10105810) is shown. The figure shows the significance of the SNPs for resistance to VNN across the whole chromosome 3 (Track 1) and across the narrow QTL region (Track 4), as well as the genes present in the region (NCBI annotation + ENSDLAG00005026832, Track 3) and expression evidence of these genes according to head kidney and brain RNA-sequencing (Track 2)

While the variants explaining > 34% of the genetic variance span a region of > 1.5 Mb, the most significant variants are located in a small region of approximately 10 kb. This region is characterized by the presence of multiple genes annotated as interferon alpha inducible protein 27-like 2A (*IFI27L2A*), and an interferon alpha inducible protein 27-like 2 (*IFI27L2*) gene (Fig. 3). However, the two available genome annotations (dlabrax2021.112 from Ensembl and GCF_905237075.1-RS_2023_02 from NCBI) for the current European sea bass genome assembly (GCA_905237075.1) show slight differences in this QTL region on chromosome 3. The 5’ untranslated region (UTR) of ENSDLAG00005028932 (Ensembl) is extremely long, while this 5’UTR sequence is replaced by a novel gene (LOC127358630) in the NCBI annotation, which is not present in the Ensembl annotation. On the contrary, a gene model present in the Ensembl annotation (ENSDLAG00005026832) is missing in the annotation of the same region by NCBI. Full-length sequence RNA-seq generated for sea bass head kidney and brain were used to establish the correct annotation for the region (Fig. 3), suggesting the presence of both LOC127358630 and ENSDLAG00005026832 genes. Therefore, a modified version of the NCBI annotation, with the ENSDLAG00005026832 added, was used for all subsequent analyses. In this annotation, there are five different interferon-induced genes (four *IFI27L2A*, and one *IFI27L2*) in the region, all located consecutively in the genome. Upstream of these genes there is a locus coding for galectin-8 (LGALS8, LOC127358615), and downstream a locus encoding a Cytosolic 5'-Nucleotidase 1A (cN-1A, LOC127358605). Indeed, 69 of the significant variants are located in the promoter-transcription start site or promoter-TSS (*n* = 16), exonic (*n* = 20), intronic (*n* = 17), intergenic (*n* = 7), and transcription termination site or TTS (*n* = 9) of the five interferon-inducible genes. The variants that showed the strongest association with resistance to VNN overlap the three most upstream of these interferon-induced genes (LOC127358630, LOC127358685, and ENSDLAG00005026832) (Fig. 3). Notably, 8 of these most significant (explaining > 34% of the genetic variance) variants are located within the 3’UTR of the ENSDLAG00005026832 gene.

### Evolutionary conservation of the QTL region

**Fig. 4:**
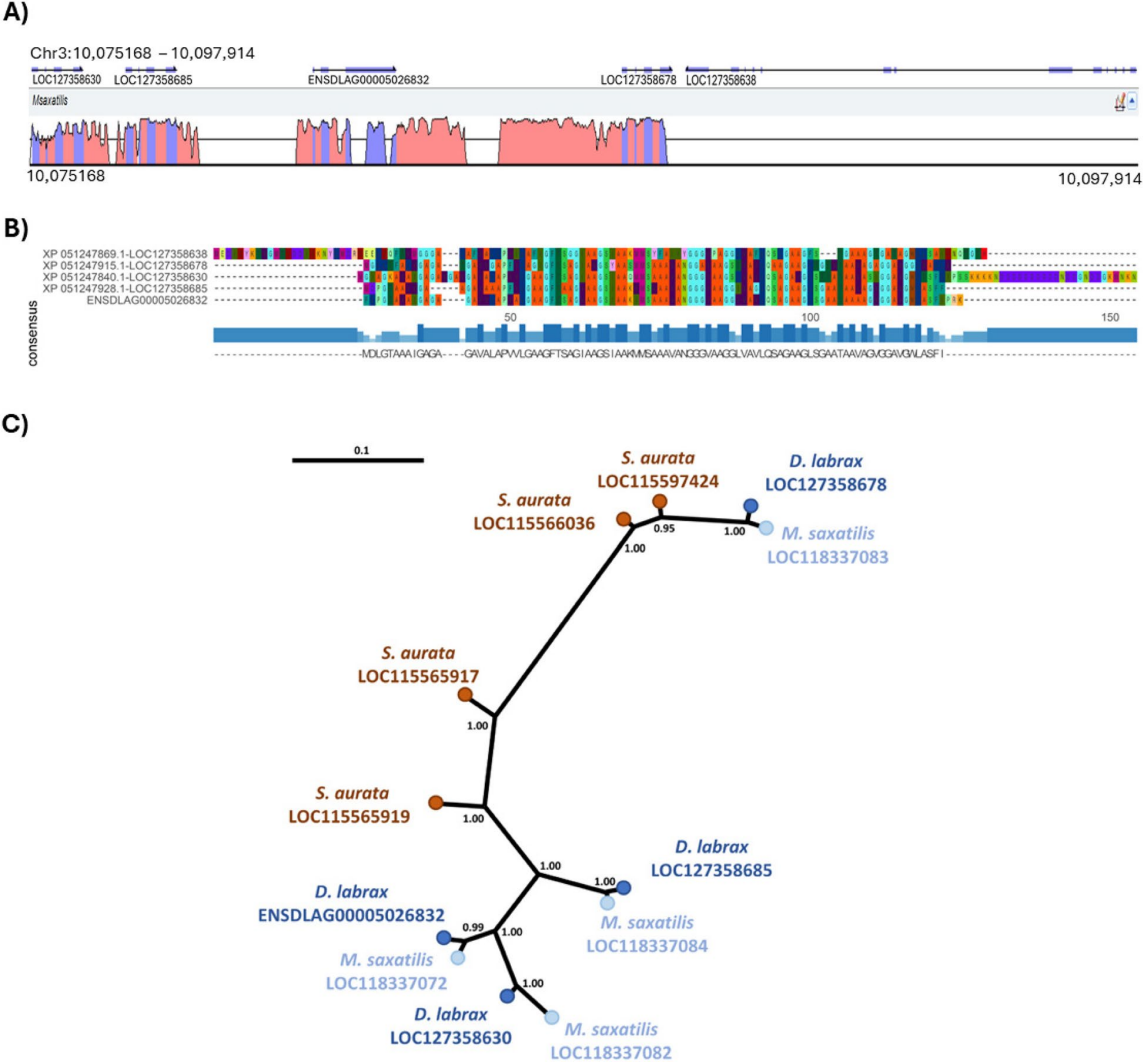
Evolutionary conservation of the major QTL region for VNN.** A** “Peaks and Valleys” graph showing the percentage of conservation of the European sea bass VNN resistance QTL region (Chromosome 3: 10,075168–10,097,914) with the homologous region in *M. saxatilis* (NCBI NW_023339873.1: 15,596,712–15,606,764), “peaks” indicate high similarity between the two species, while “valleys” indicate low similarity. **B** Multiple sequence alignment plot of the Amino acid sequences of the five interferon-induced genes in the VNN resistance QTL region of European sea bass, where letters correspond to the standard abbreviation of amino acid residues in the peptide sequences, while “-” represents a gap in the alignments. **C** IFI27L2-like gene tree reconstructed using Bayesian inference. GeneIDs are reported at the branch tips for consistency throughout the document, but the corresponding coding mRNA sequences were used for the analysis (*D. labrax*: LOC127358678—XM_051391955.1, LOC127358685—XM_051391968.1, LOC127358630—XM_051391880.1; *S. aurata*: LOC115566036—XM_030391784.1, LOC115597424—XM_030443305.1, LOC115565917—XM_030391575.1, and LOC115565919—XM_030391576.1; *M. saxatilis*: LOC118337083—XM_035673933.1, LOC118337072—XM_035673917.1, LOC118337084—XM_035673934.1, LOC118337082—XM_035673932.1). Numbers at nodes are posterior probability values

To discard potential misassemblies and confirm the existence of multiple copies of interferon-induced genes (*IFI27L2A* and *IFI27L2*), the European sea bass major QTL region was compared with homologous genomic regions in the striped sea bass (*Morone saxatilis)*, a closely related species of the same family Moronidae, and the gilthead sea bream (*Sparus aurata)*, a more distantly related species. Both species have a cluster of four copies of interferon-induced genes (three *IFI27L2A* and one *IFI27L2*), and similar to European sea bass, this gene cluster is flanked by galectin-8 upstream and cytosolic 5'-nucleotidase 1A downstream. Comparison of the homologous genomic regions in the striped bass and the European sea bass showed high sequence conservation and consistent gene order, apart from the absence of a homologue for LOC127358638 in *M. saxatilis* (Fig. 4A).

Sequence alignment of the five interferon-induced European sea bass genes/proteins (four *IFI27L2A*, and one *IFI27L2*) revealed that this LOC127358638 has diverged significantly of the other four interferon-induced genes in the cluster, which had relatively high sequence similarity (Fig. 4B). Considering the high sequence divergence, and the lack of a homologous gene in *M. saxatilis*, this gene was excluded from phylogenetic analyses since it would significantly disrupt protein alignments. While not as extreme, the other four sea bass interferon-induced genes also showed substantial sequence divergence, with LOC127358678 being quite different from the three upstream interferon-induced genes (Fig. 4B).

To reconstruct the evolution of the other four IFI27L2 genes in *D. labrax*, their coding mRNA sequences were aligned against those of *M. saxatilis* and *S. aurata*, which confirmed that these European sea bass interferon-induced genes (LOC127358630, LOC127358685, ENSDLAG00005026832, LOC127358678) have one-to-one orthologues in the striped sea bass genome (Fig. 4C), suggesting that duplication events occurred in the common ancestor of the two species. These three genes formed a relatively tight phylogenetic cluster, reflecting their high sequence similarity.

### Expression QTL analysis shows evidence of genetic variants controlling the expression of the three upstream IFI27L2 genes

Brain and head kidney RNA-Seq data of 110 mock and 214 VNN-challenged fish from the same families used for the larger GWAS disease challenge were produced. Of these fish, 322 had acceptable high-quality ~ 30 K SNP array genotypes and hence imputed to whole-genome genotypes based on the whole-genome resequencing of their parents and 40 sibs. Animals were classified based on their QTL genotypes, and differential expression analyses between individuals homozygous for the resistant allele and homozygous for the susceptible allele (based on the three most significant SNPs) revealed differences for three interferon-induced genes (LOC127358630, LOC127358685, and ENSDLAG00005026832) (Table 2). However, these differences were unstable (i.e. none of the three genes showed significant differences across the two conditions and the two tissues). Nonetheless, the homozygous “resistant” group showed higher expression at all three loci in at least one tissue/condition with statistically significant fold changes (FC) ranging from 1.58 to 2.57 (Table 2).

**Table 2:** Differential expression between homozygous resistant (RR) and homozygous susceptible (SS) European sea bass fish based on the major VNN resistance QTL genotype

Tissue	Condition	GeneID	FDR *p*-value	Log2 FC
**Brain**	**Mock**	LGALS8	1	-
		LOC127358630	2.49E − 23	1.21
		LOC127358685	0.99	-
		ENSDLAG0005026832	1	-
		LOC127358678	1	-
		LOC127358638	1	-
	**VNN**	LGALS8	0.86	-
		LOC127358630	0.97	-
		LOC127358685	0.13	-
		ENSDLAG0005026832	0.56	-
		LOC127358678	0.49	-
		LOC127358638	0.72	-
**Head kidney**	**Mock**	LGALS8	1	-
		LOC127358630	0.02	1.00
		LOC127358685	0.02	1.36
		ENSDLAG0005026832	8.27E − 05	0.66
		LOC127358678	1	-
		LOC127358638	1	-
	**VNN**	LGALS8	0.97	-
		LOC127358630	0.97	-
		LOC127358685	8.07E − 06	0.91
		ENSDLAG0005026832	2.85E − 11	0.70
		LOC127358678	0.99	-
		LOC127358638	0.90	-

**Fig. 5:**
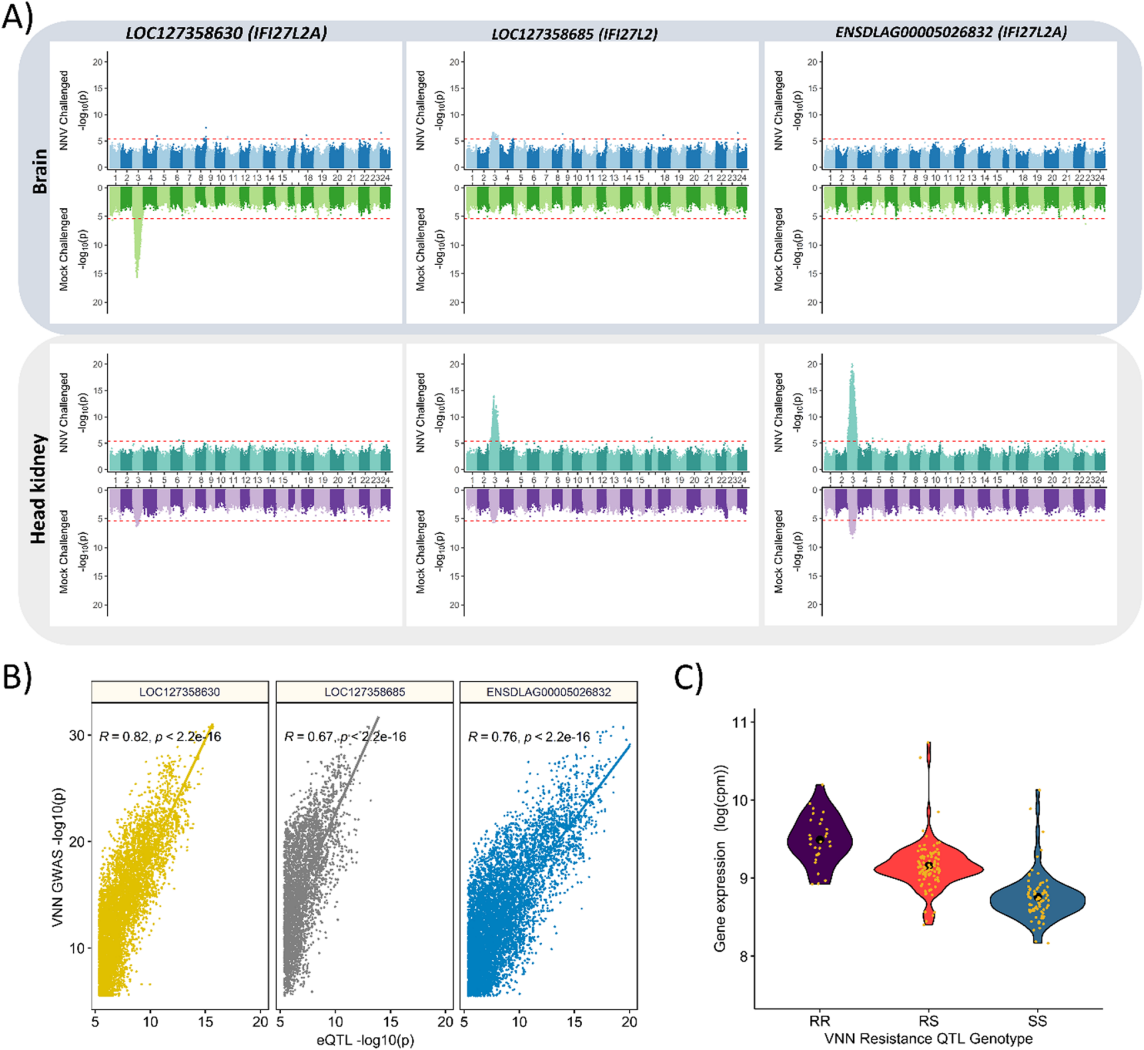
Association between IFI27L2 gene expression in European sea bass and the QTL genotype.** A** Expression QTL analysis for the three IFI27L2/2A genes in the proximal region of the VNN resistance QTL in chromosome 3. **B** Pearson correlation between the expression QTL analysis *p*-values for the three proximal IFI27L2/2A genes and the *p*-values for resistance to VNN based on the whole-genome GWAS. **C** Violin plot showing the gene expression of IFI27L2/2A in animals with different VNN resistance QTL genotypes: RR (two resistant alleles), RS (one resistant and one susceptible allele), SS (two susceptible alleles)

Consistently, global correlation analyses showed strong correlations (*r* = 0.2–0.49; *p* < 0.005) between the expression of the three interferon-induced genes and predicted VNN resistance breeding values. Noteworthy, the correlations of the three genes varied between conditions and tissues with only LOC127358685 and ENSDLAG0005026832 showing consistent significant correlations in the head kidney in the mock and VNN-challenged fish. Expression QTL (eQTL) analyses for the three interferon-induced genes revealed a clear association between the SNPs in the VNN QTL region and the expression of these genes (Fig. 5A). As observed in the differential expression analyses and correlation analyses, the eQTL results varied across conditions and tissues; nevertheless, the association between QTL genotypes and the expression of the three interferon-induced genes was unequivocal. Notably, the genetic variants that were highly significant in the eQTL analysis overlapped with those described above associated with VNN resistance (Fig. 5B). In all cases, the minor frequency “alternative” allele was associated with higher gene expression as well as better survival after VNN infection, and it showed a clear significant (*p* ≤ 2.9E07) additive effect, with heterozygous animals showing intermediate expression of *IFI27L2/2A genes* (Fig. 5C).

### Analysis of sequence variants in putative regulatory regions

**Fig. 6:**
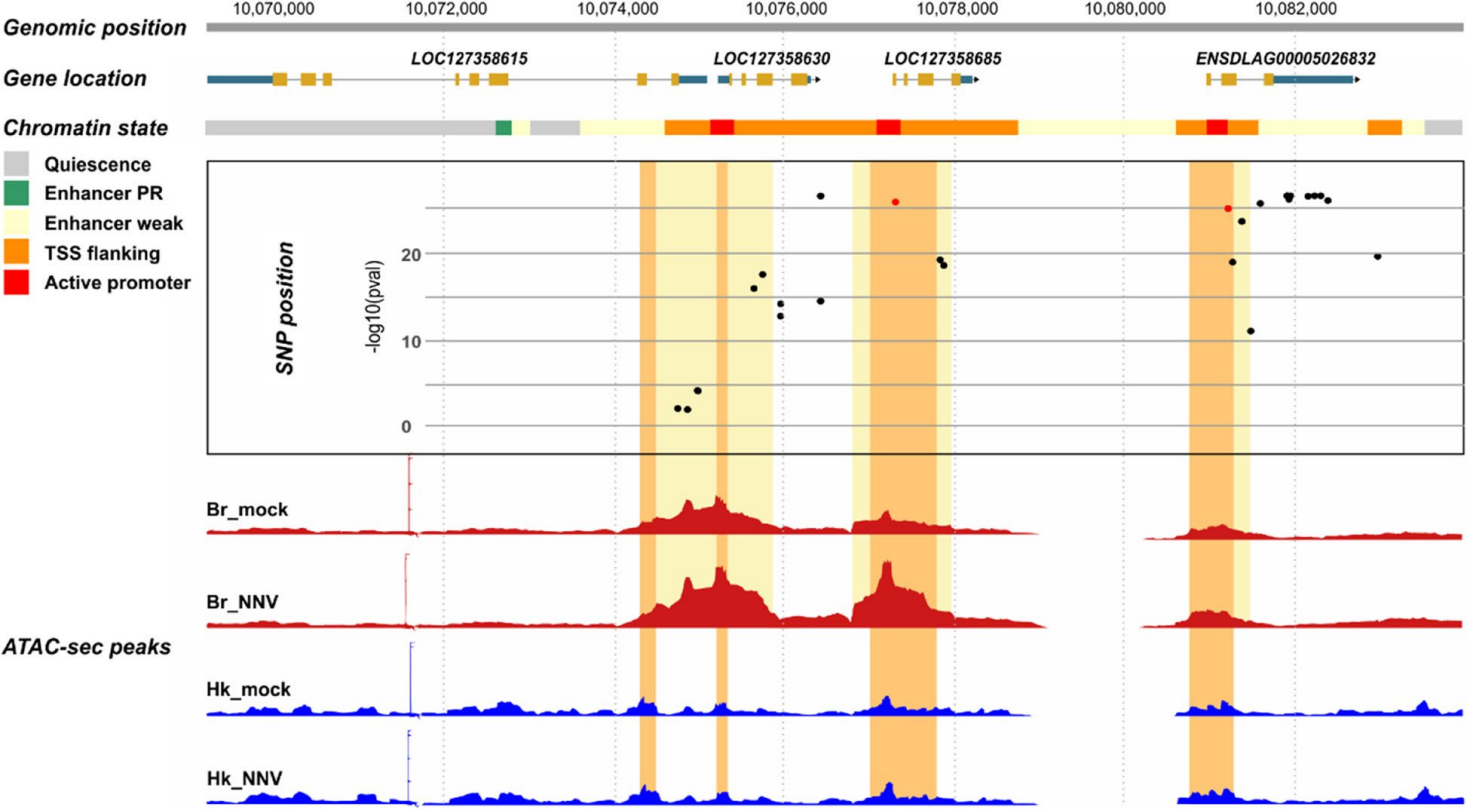
Regulatory landscape of the VNN resistance QTL region in the head kidney and the brain. The figure shows the VNN resistance QTL region, the position of the four expressed IFI27L2 genes, the chromatin state based on ATAC-seq and ChIP-seq assays, VNN resistance GWAS *p*-values for the SNPs in the regions, and chromatin accessibility peaks in mock and VNN challenges brain (Br) and head kidney (Hk)

A subset of 20 fish (10 mock- and 10 VNN-infected) of the ones with RNA-seq data were used to assess chromatin accessibility using ATAC-seq in brain and head kidney tissues. In the major QTL region, four open chromatin regions were identified in the head kidney and three in the brain, with substantial overlap between the tissues (Fig. 6). No differences were observed between mock and VNN-challenged samples. Additionally, active regulatory regions were predicted using both ATAC-seq and ChIP-seq for three different histone marks, finding overlap between the open chromatin regions and active regulatory elements (TSS flank and TSS active) (Fig. 6). Two single-nucleotide variants, located in CHR3:10,077,301 and CHR3:10,081,209, showing high association with survival to VNN and with the expression of the three upstream *IFI27L2* genes, were located within the putative regulatory regions of LOC127358685 and ENSDLAG00005026832, respectively, which are shared between brain and head kidney (Fig. 6).

To evaluate the potential effect of these two variants on transcriptional regulation, the variant effects on transcription factor binding significant SNPs were predicted using FABIAN-variant. For SNP CHR3:10,077,301, the alternative (resistant) allele was predicted to lead to a transcription factor binding site (TFBS) gain for human KLF transcription factor 15 (KLF15, ENSG00000163884) with a high score of 0.8146 on a 0–1 scale (Additional file 1: Fig. S2). For CHR3:10,081,209, two TFBS gains were predicted for the alternative (resistant) allele, for zinc finger protein 140 (ZNF140, ENSG00000196387) and TEA domain transcription factor 4 (TEAD4, ENSG00000197905), respectively, with a score of 0.9808 and 0.9421 (Additional file 1: Fig. S2). Human ZNF140 does not have an orthologue in the European sea bass, while there are 1:1 orthologues for KLF15 (ENSDLAG00005009091) and TEAD4 (ENSDLAG00005022250). Sequence similarity at the protein level was high for TEAD4 (on average > 80%) and very high for the TEA domain. For KLF15, similarity was moderate (on average > 50%), although the DNA binding domain was highly conserved.

### Distribution of allele frequencies across natural wild populations

The two variants were also analysed in terms of allele frequencies in wild populations of sea bass from different geographical areas (i.e. Atlantic Ocean, Western Mediterranean Sea, Eastern Mediterranean Sea), which are known to be genetically divergent and show differential resistance to VNN; Eastern Mediterranean populations have a significantly higher survival (up to three times) upon experimental infection with VNN compared to fish of Atlantic and Western Mediterranean origin [4]. Analysis of published pool-sequencing WGS data [29] allowed the estimation of allele frequencies for the two variants (Table 3). The minor allele variants associated with disease resistance and higher expression of interferon-induced genes showed significantly higher frequency in Eastern Mediterranean population samples (CHR3:10,077,301 *p* < 0.00001; CHR:10,081,209 *p* < 0.01), with the difference substantially larger for CHR3:10,077,301 (0.45 vs 0.08). Similarly, frequencies of four variants (CHR3:10,081,901, CHR3:10,081,923, CHR3:10,082,148 and CHR3:10,082,380) located in the 3’UTR of ENSDLAG00005026832 (*IFI27L2A*) gene were evaluated in three wild European seabass populations using whole-genome sequence genotype data (unpublished). The allelic frequency of the resistant allele of all four variants was higher (0.75–0.82) in the VNN-resistant population (East Mediterranean) as compared to the Atlantic (0.08–0.35) and West Mediterranean (0.05–0.25) populations which are VNN-susceptible populations (Additional file 1: Table S1).

**Table 3:** Minor allele frequencies for the two main VNN resistance candidate SNPs

*Variant*	*ATL*	*WMED1*	*WMED2*	*EMED1*	*EMED2*
CHR3:10,077,301	0.21	0.15	0.25	0.55	0.77
CHR3:10,081,209	0.05	0.07	0	0.12	0.13

The minor alleles are the VNN resistance alleles. ATL—Atlantic population (susceptible), WMED1 & WMED2—Western Mediterranean populations (susceptible); EMED1 & EMED2 (resistant).

## Discussion

Our results confirm that VNN resistance has moderate to high heritability (0.35 to 0.45) in farmed European sea bass, with slightly higher values than suggested by previous studies [9–13], and therefore, there is great potential for selective breeding to produce fish that are more resistant to this disease, reducing VNN’s economic impact and improving fish welfare. Our GWAS results also confirmed the presence of a very strong QTL for VNN resistance, located on Chromosome 3 of the European sea bass genome, consistent with similar studies in different farmed sea bass populations [9, 11, 12]. The most significant SNPs in the QTL explained up to 38% of the total genetic variance, which is comparable to the report by Vela-Avitúa et al. where the most significant SNP explained 33.33% of VNN resistance genetic variance [12]. However, Griot et al. reported a substantially lower genetic variance (9%) explained by the most significant markers [9]. This difference could depend on the infection challenge model, while in both our study and Vela-Avitúa et al., fish were infected via intraperitoneal/intramuscular injection [12]. Griot et al. performed an immersion challenge [9], which could have resulted into different infection dynamics as immersed animals have additional physical layers of immune protection against the virus including the scales, skin, mucus layer, epithelial layer of the gills, and alimentary canal which all play fundamental roles in preventing pathogen entry into the body of the animal [30, 31]. Another potential explanation is the difference in the crossing scheme, as Griot et al. used four full-sib backcross families (Eastern and Western Mediterranean sires, Western Mediterranean dams) and two farmed populations for their analyses [9]. The relatively small number of families of diverse origin could explain the relatively low genetic variance explained by associated markers in the latter study.

As already mentioned, in all aforementioned studies, the precise identity of the putative causative variants remained elusive. Using imputed WGS data, Delpuech et al. identified four VNN-resistance QTLs on LG12 (presently Chromosome 3) [14]. The most significant one was centred on a region of approximately 50 kb (LG12:8,750,00–8,800,000), which includes two protein-coding genes (ZDHHC14 and IFI6/IFI27-like). Highly significant SNPs were represented by a non-synonymous substitution on ZDHHC14 and a non-coding polymorphism (the most significant one) 3.7 kb downstream of IFI6/IFI27-like. Additionally, all previous studies on the genetic assessment of VNN resistance in European sea bass [9, 11, 12, 14] are based on a previous genome assembly [15], which was produced using short-read sequencing technologies, resulting in a highly fragmented assembly with several sequence gaps. Here, we used for the first time a novel, highly contiguous genome assembly built using long sequence reads. In fact, analysis of the candidate QTL region in the old genome assembly used by Delpuech et al. shows that there are only two IFI6/IFI27-like genes in the candidate region [14], which likely correspond to LOC127358685 and ENSDLAG00005026832 in the novel assembly. Not surprisingly, in the old assembly there is a sequence gap between the ZDHHC14 locus and the two IFI6/IFI27-like genes in the region, the three missing IFI27-like genes that are part of the cluster in the new assembly are found in unplaced contigs in the previous version of the genome. In the novel assembly, the ZDHHC14 gene is located in chromosome 3, but at several Mb of distance from the region we identified as the most significantly associated with VNN resistance. All this evidence suggests that the previous discordance regarding the position of the QTL could be explained, at least in part, by the lower quality of the previous genome assembly.

In our study, whole-genome sequencing and imputation based on the novel genome assembly provided a clearer picture of the genetic background of VNN resistance in farmed sea bass when compared to the SNP array. The position of the major QTL was refined and narrowed to a relatively small genomic region, with the most significant SNPs overlapping two *IFI27L2A* genes, and one *IFI27L2* gene. The most significant SNP reported by Delpuech et al. is located 3.7 kb downstream of one of these genes (LOC127358685) [14], putatively overlapping with the ENSDLAG00005026832 locus in the novel assembly. Co-localization of the most significant variants from GWAS and eQTL analysis strongly supports the hypothesis that the causative SNP(s) is(are) regulatory variant(s), significantly altering the expression of *IFI27L2* and the two *IFI27L2A* genes [32].

Human *IFI27L2A* encodes for interferon alpha-inducible protein 27-like protein 2A, which is an interferon-stimulated gene (ISG) and is sometimes referred to as ISG12, while *IFI27L2* which is also referred to as *ISG12B,* encodes for the interferon alpha-inducible protein 27-like protein 2. Upon detection of viral infection, host cells release interferons that in turn upregulate the expression of several ISGs, which combat the infection through viral degradation and the inhibition of viral replication [33]. Interestingly, in mammals *IFI27L2A* has antiviral properties in the central nervous system [34]. Lucas et al. demonstrated, using a mice *IFI27L2A* knockout, that *IFI27L2A* protected mice against lethal West Nile virus (WNV) infection by significantly restricting replication of this virus in the different tissues of the central nervous system [34]. Recently, a cluster of eight *IFI27L2A* homologues (ISG12.1–8) has been reported on zebrafish chromosome 13, and ISG12.1 was shown to suppress virus replication via targeting viral phosphoprotein [35], suggesting a conserved antiviral role of *IFI27L2A* in fish. *IFI27L2A* is upregulated in response to VNN in European sea bass, and in fact an IFI27L2-encoding gene (FN665389.1), corresponding to LOC126358685, has been used since 2010 in several studies to monitor the response to either VNN infection or vaccination in this species [36]. This is consistent with studies in other teleost species, where *IFI27L2A* was upregulated in response to VNN infection [37]. The upregulation of three (LOC127358630, LOC127358685, ENSDLAG00005026832) of the five interferon-induced genes present in the European sea bass in response to VNN, and the association of the VNN-resistance alleles with higher expression of these genes, strongly suggest that *IFI27L2A*/ *IFI27L2* could be the causative genes underlying the major QTL for resistance to VNN. While the observed pattern appears to be tissue-and condition-specific for the three *IFI27L2A/ IFI27L2* genes, this suggests that resistance is favoured by “constitutive” higher levels of mRNA of the genes.

The observed complex pattern of tissue- and condition-specific expression is not surprising, since duplicated loci are known to evolve through neo-functionalization and sub-functionalization [38], leading to different tissue- and stimulus-specific patterns. Moreover, regulation of gene expression is a complex mechanism, involving the cooperation of multiple transcription factors and co-factor/co-repressors that recognize different DNA motifs (regulatory elements) located mostly in the proximity of the regulated gene, but that can also be located at a substantial distance of the gene promoter [39]. Finally, evidence from single-cell functional genomic studies has shown that “bulk” analysis on whole tissues and organ samples offers a coarse view of gene regulation [40]. Albeit with a very limited sample size, single-cell midbrain transcriptomics in another VNN-susceptible species, the orange spotted grouper *Epinephelus coioides* suggested that specific cell types are involved in the antiviral response and only certain neuronal cell lineages are damaged by the virus [41]. While eQTL analyses using single-cell RNA-seq data are still prohibitively expensive for non-model species, they could vastly help us understand the observed expression patterns and genetic regulation of the different European sea bass IFI27L2A genes.

### Two SNPs in the promoter regions of IFI27L2A genes are strong candidate variants for the VNN resistance QTL

The combination of disease phenotypes, WGS genotypes, eQTL-level RNA sequencing, and ATAC-seq and ChIP-seq, significantly reduced the number of putative causative variants. In particular, the use of chromatin accessibility, an essential feature of regulatory elements, allowed us to narrow the number of candidates to two SNPs. Both are located on putative promoter regions of *IFI27L2A* genes and are predicted to result in novel binding sites for two different transcription factors, KLF15 and TEAD4, which are involved in regulating gene expression in immune cells [42, 43]. KLF15 is also an important stress-induced gene in fish [44], and experimental infection with VNN in European sea bass has been reported to activate a strong stress response [45]. While evidence of the effect of genetic variants on TFBS predicted using human/mouse data should be taken with caution, high sequence conservation at the amino acid level frequently suggests that DNA–protein interactions are conserved. The two identified SNPs are strong causative candidates for the VNN-resistance QTL.

Population genomics data further supports the role of these two variants in resistance to VNN. It has been consistently shown that European sea bass individuals originating from Eastern Mediterranean populations have a significantly higher survival (up to three times) upon experimental infection with VNN compared to fish of Atlantic and Western Mediterranean origin [4, 8]. Our analysis of allele frequencies revealed that alternative alleles for both SNPs have a significantly higher frequency in Eastern Mediterranean populations compared to Atlantic and Western populations, further corroborating our findings. However, the difference is much more pronounced for the variants in CHR3:10,077,301, with a considerably higher frequency of the resistant allele, which suggests this variant is the strongest candidate causative mutation for the major QTL for resistance to VNN.

This CHR3:10,077,301 variant is located in the promoter of LOC127358685, generating the KLF15 binding site, and shows association with the expression of the three interferon-induced genes (LOC127358685, LOC127358630, and ENSDLAG00005026832) in both tissues. While it is located in the promoter region of LOC127358685, the variant could also modulate the expression of the other two genes, as promoters are known to often act also as enhancers (termed as Epromoters), exerting their effects on other loci [46, 47]. However, we should not exclude the possibility that there could be other candidate variants, or even additional variants, that are involved in the genetic basis of resistance; massive parallel reporter assays (MPRAs) have shown that at least 17.7% of eQTLs have multiple, tightly linked causal variants [48]. Nevertheless, the SNP in CHR3:10,077,301 is a strong candidate to regulate resistance to VNN in European sea bass via modulation of the expression of the interferon-induced gene LOC127358685.

It is also worth highlighting that a significant number (*n* = 8) of the identified variants as having the strongest association with resistance to VNN infection are located within the 3’UTR region of the ENSDLAG00005026832 (*IFI27L2A*). These variants are also strongly associated with modulating the expression of the ENSDLAG00005026832 gene, especially in the head kidney. Interestingly, the frequency of the VNN-resistance alleles of four of these variants were remarkably higher in the VNN-resistant population (Eastern Mediterranean) compared to the populations known to be susceptible to VNN infection. Given these results, it is plausible also to speculate that VNN resistance in European seabass is potentially regulated via modulation of 3’UTR post-transcriptional activities such as micro-RNA (miRNA) and RNA-binding protein (RBP) mediated decay [49] of ENSDLAG00005026832 (*IFI27L2A*) mRNA.

## Conclusions

Precise identification of causal variants for complex traits remains a major challenge in human and animal genomics, but integration of multiple functional evidence has already shown a great potential to finely map causative mutations. The integration of phenotyping, genome-wide genotyping and multiple functional genomics technologies assessing both the coding and non-coding regions of the genome have allowed us to identify the putative causative loci and genes underlying a major QTL for resistance to VNN in European sea bass. This study paves the way for biotechnological applications to generate fully VNN-resistant animals in European sea bass or other susceptible species; tools such as marker-assisted selection and genome editing that can exploit the discovered loci to increase VNN resistance in aquaculture species, improving animal welfare and the sustainability of food production.

## Methods

### Fish population generation and VNN challenge test

The experimental fish were produced in January 2020 using a commercial, NNV-free tested, broodstock from Valle Cà Zuliani Società Agricola srl (Pila di Porto Tolle, Rovigo, Italy). Dams were subjected to ovarian biopsy and those with a suitable stage of development of eggs were hormonally injected with LH-RHa (luteinizing hormone releasing-hormone analogue, 10 μg/kg body weight) and subsequently stripped 72 h after the injection. Upon striping, eggs were immediately mixed with previously stripped and preserved sperm [50] and seawater, then moved to the hatchery tanks after 5–6 min (2000-L tanks with conical bases). The mating scheme was based on a full-factorial design (25 dams × 25 sires). Eggs were mixed and distributed in four rearing tanks. At an age of 240 days post-hatching and an average weight of 13 g, approximately 1029 fish were transferred to the Istituto Zooprofilattico Sperimentale delle Venezie (IZSVe, Legnaro, Padova, Italy) and equally distributed into three close-system 2500-L tanks. Each tank was filled with artificial saltwater (30‰ salinity, temperature 21 ± 1°C, oxygen 6 ppm) and exposed to artificial photoperiod (10 h of light, 14 h of darkness). After an acclimation period of 14 days, fish were individually tagged with passive integrated transponder (PIT) tags and subjected to a VNN challenge test through intramuscular injection of 0.1 mL of a 1:100 dilution of the viral suspension (RGNNV 283.2009, stock titre 10^8.30^ TCID_50_ per mL) [51]. The procedure was performed on anaesthetized animals (MS-222, 30 ppm). Water temperature was increased to 25 ± 1°C, while the other parameters (salinity, oxygen, photoperiod) were the same as in the acclimation period. Mortality was recorded twice a day. The challenge test ended after 29 days, when mortality returned to baseline levels. Resistance to VNN infection was defined as binary survival (BS, 0 survivor fish, 1 dead fish) and days to death (DD).

An additional group of fish (*n* = 324) from the same factorial crossing (i.e. full and half-sibs of the challenged animals) were exposed to VNN to assess the transcriptomic response to the infection. Of these 324 fish, 214 were VNN challenged through intraperitoneal injection of 0.1 mL of a 1:100 dilution of the viral suspension (RGNNV 283.2009, stock titre 10^8.30^ TCID_50_ per mL [46], while 110 were mock-challenged through injection of Dulbecco’s modified Eagle’s medium (DMEM), the cell culture medium where the virus was cultured. For each fish, brain and head kidney were sampled at 48 h post-challenge for RNA sequencing (peak of the immune response, determined based on a preliminary transcriptomic study at 6, 12, 24, 48, and 72 h post-challenge on the same population; data not shown).

Fin clips of all the animals, including the parents, were collected and individually preserved in 70% ethanol and stored at 4°C for genotyping.

### DNA extraction and genotyping

The genomic DNA of all the fish used in this study (including 50 parents and 1353 offspring) was extracted from fin clips using Chelex method, employing Chelex as the extraction medium [52] and genotyped on the bi-species MedFish SNP array [53] containing 29,888 European sea bass SNPs, which was performed by IdentiGen Ltd (Dublin, Ireland). The array intensity files were imported to the Axiom analysis Suite v4.0.3.3 software for quality control (QC) assessment and genotype calling. SNPs were called following the default recommendations for diploid species (call rate threshold > 97%). A total of 1362 fish (97%) and 28,404 high-quality SNPs (95%) were retained for further analysis. The samples that passed QC included all 50 parental fish, 990 VNN resistance-phenotyped animals, and 322 VNN transcriptome challenge samples.

All parents (25 dams and 25 dams) and 40 resistance-phenotyped offspring were whole-genome sequenced by Novogene (Cambridge, UK) to obtain whole-genome genotypes for imputation. Genomic DNA was extracted from fin clip tissue using the salt-extraction method [54]. Subsequently, DNA sequencing libraries were prepared using Illumina TruSeq DNA Preparation Kits at the facilities of Novogene and then sequenced on a NovaSeq 6000 platform (Illumina, California, San Diego, USA) as 150PE reads to a sequencing depth of 15X. Raw sequence reads were assessed for sequencing quality using FastQC v0.11.9 [55], and consequently trimmed of any sequencing adaptors and low-quality sequences using Trimmomatic v0.32 [56]; high-quality reads of length ranging from 36 to 150 bp were retained for variant calling. Burrows-Wheeler Aligner (BWA) v0.7.187 [57] was then used to align the clean reads to the European sea bass genome dlabrax2021 (GCA_905237075.1) downloaded from Ensembl (http://ftp.ensembl.org/pub/release-107/fasta/dicentrarchus_labrax/dna/Dicentrarchus_labrax.dlabrax2021.dna.toplevel.fa.gz). Samtools v1.15 [58] and BCFtools [58] were subsequently used for genotype calling.

### Genotype quality control assessment and cleaning, parentage assignment, and imputation

SNP array genotype data was further filtered using PLINK v1.9 [59], removing SNPs with genotype call rate across samples (geno) < 95%, minor allele frequency (MAF) < 5%, and significantly deviating from Hardy–Weinberg equilibrium (HWE) < 1E − 4; samples missing > 10% of the genotypes were also removed from the analysis. After QC, 1312 animals and 24,740 SNP markers were retained. Principal component analysis (–pca) was performed in PLINK v1.9 using all remaining SNPs to assess the genetic structure of the study population. Parentage assignment was performed using APIS package v1.0.1 [60] in R v4.1.1 through estimation of average Mendelian allele transmission probability between parents (sires and dams) and offspring using all 24,740 SNPs, allowing an assignment error rate of 1%.

WGS genotypes were filtered using vcftools v0.1.13 [61], removing variants (SNPs and Indels) with more than two alleles (–max-alleles 2), genotyped in less than 50% of the samples (–max-missing 0.5), with genotype quality < 30 (–minQ 30), low coverage < 8 reads (–minDP 8), or very high coverage > 31 reads (–maxDP 31.05), and with minor allele count < 3 (–mac 3). Additionally, variants strongly deviating from Hardy–Weinberg equilibrium (–hwe 1E − 7) in the parental population were also removed using PLINK v1.9 [59]. After QC, a total of 8,368,178 variants (i.e. 7,538,357 SNPs and 829,821 indels) were retained for further analyses.

Fimpute version 3 [62] was used to perform genotype imputation for all the offspring (genotyped using the SNP array, 24,740 SNPs) using the parents as reference (genotyped using WGS, 8,368,178 SNPs), resulting in a dataset of ~ 8.4 M variants.

### Survival analysis and estimation of genetic parameters

Survival analyses were performed for the VNN resistance phenotyped offspring using the Survminer v0.4.9 package [63] in R v4.1.1. Bivariate restricted maximum likelihood (–reml-bivar) analyses were performed in GCTA version 1.93.2beta [64] to estimate genomic parameters (heritability and genetic correlations) of VNN resistance traits using SNP array and WGS imputed genotypes. VNN resistance phenotypes were preadjusted for population genetic substructure using the first and second principal components obtained using PLINK v1.9 [59] as described above.

### Genome-wide association study

Genome-wide association study (GWAS) analyses were performed through single marker association testing using the mixed linear model leaving-one-chromosome-out (LOCO) implemented in GCTA v1.94.1 (–mlma-loco) [64]. The following linear mixed model was fitted for the association analysis:

$$y_{ij}=u+b_jx_{ij}+a_i+\varepsilon_{ij}$$

where *y*_*ij*_ was the pre-adjusted VNN resistance phenotypic value (binary survival or days to death) of the *i*th individual, *b*_*j*_ is the allele substation effect of the *j*th variant, *x*_*ij*_ are the *j*th variant genotype classes coded as 0, 1, 2 (number of copies of the minor allele), *a*_*i*_ is the random additive polygenic effect (breeding value) of the *i*th individual, and *ε*_*ij*_ is the random residual effect of the *i*th individual with the *x*_*ij*_ genotype of *j*th SNP. The random additive polygenic effects (a) are assumed to be independent and follow a normal distribution $a \sim N\left(0,{\varvec{G}}{\sigma }_{a}^{2}\right)$ where ${\sigma }_{a}^{2}$ is the additive polygenic effect variance of the trait in the study population and *G* is the genomic relationship matrix between all individuals constructed in GCTA v1.94.1 (–make-grm) using the genotypes of all variants except those located on the chromosome of the variant under consideration. The random residual effects (ε) were also assumed to follow a normal distribution $\upvarepsilon \sim \boldsymbol{ }N(0,\boldsymbol{ }{\varvec{I}}{\sigma }_{\upvarepsilon }^{2})$ where ${\sigma }_{\upvarepsilon }^{2}$ is the residual variance and ***I*** is an identity matrix of *n* × *n* dimensions (where *n* is the number of individuals in the analysis).

*P*-values for the variants from the analysis were adjusted for multiple testing using the Benjamini-Yekutieli (BY) method [65] and the variant was considered significantly associated with the trait at an adjusted *P*-value < 0.01. Phenotypic variance explained by a single variant was calculated as $v_p=\frac{2pq\beta^2}{\sigma_p^2}\times100\%$, while additive genetic variance explained by each variant was calculated as $v_p=\frac{2pq\beta^2}{\sigma_a^2}\times100\%$, where *p* and *q* are the minor and major allelic frequencies of the variant, *β* is the allele substitution effect, ${\sigma }_{p}^{2}$ and ${\sigma }_{a}^{2}$ are the overall phenotypic variance and additive genetic variance of the trait in the studied population.

### RNA sequencing, differential gene expression and expression QTL analyses

Total RNA was extracted from the head kidney and brain of the 110 mock challenged fish and 214 VNN infected fish of the secondary experiment using RNeasy Mini Kit (Qiagen, Hilden, Germany) according to the instructions of the manufacturer. RNA concentration and quality was assessed using an Agilent 2100 Bioanalyzer with the RNA Nano 6000 kit (Agilent Technologies, Santa Clara, CA, USA), and only RNA samples with RIN > 7 were processed for subsequent cDNA library construction and sequencing. cDNA libraries were constructed using the QuantSeq 3′ mRNA-Seq Library Prep Kit FWD (Illumina, Lexogen GmbH, Vienna, Austria) following the manufacturer’s instructions, and sequenced on a NovaSeq 6000 sequencing platform (Illumina, California, San Diego, USA) as 75 bp single-end reads. Raw read sequence data was assessed for sequencing quality using FastQC v0.11.9 [55], and low-quality reads and residual adaptors were removed using BBDuk v38.84 [66]. High-quality reads were then mapped against the European sea bass genome (GCA_905237075.1) using the short-read aligner STAR v2.7.3a [67]. Gene counts were produced using featureCounts v2.0.0 [68] and the annotation of the European sea bass genome, with the option “–primary” that counts the primary alignment of multimapping reads (in addition to uniquely mapped reads).

Differential expression between susceptible and resistant QTL genotypes was performed with the R package DESeq2 v1.38 [69] using the standard pipeline. Briefly, separately for each tissue (i.e. brain and head kidney) and condition (i.e. mock and VNN-challenged animals), the relevant samples were extracted from the count table and imported in DESeq2 with the function “DESeqdataSetFromMatrix”; in order to remove uninformative genes that could contribute to background noise [70], the matrix of gene counts was filtered to keep genes with > 20 raw reads in total across all samples. Then, the filtered count table was normalized using the “varianceStabilizingTransformation” function and differential expression analysis was performed using the function “DESeq” by contrasting the resistant vs susceptible genotypes and performing a Wald significance test with adjusted *p*-value threshold < 0.05.

Additionally, using the VNN resistance phenotyped fish as reference, the genomic best linear unbiased prediction (GBLUP) approach implemented in GCTA v1.94.1 was used to compute genomic estimated breeding values (GEBVs) for resistance to VNN for the transcriptome immune challenge experiment (not phenotyped) using the linear mixed model bellow:

$$y=1_n\mu+Zg+e$$

where *y* is a vector of VNN susceptibility or resistance phenotypes (pre-corrected for genetic structure), *1n* is a vector of ones, *μ* is the overall average, *g* is a vector of random additive polygenic effects (genomic breeding values) assumed to follow a normal distribution $g \sim N\left(0,{\varvec{G}}{\sigma }_{g}^{2}\right)$ where *G* is the relationship matrix between all individuals constructed in GCTA v1.94.1 (–make-grm) using genome-wide markers, while ${\sigma }_{g}^{2}$ is the additive genetic variance of the trait, *Z* is a design matrix linking the random additive polygenic effects (genomic breeding values) in *g* to the phenotypes in *y*. And *e* is a vector of random residual values assumed to follow a normal distribution $e \sim \boldsymbol{ }N(0,\boldsymbol{ }{\varvec{I}}{\sigma }_{\text{e}}^{2})$ where ***I*** is an identity matrix and ${\sigma }_{\text{e}}^{2}$ is the residual variance. Pearson’s correlations were then computed between gene expression log_2_CPM values of expressed genes and VNN resistance GEBVs for mock challenged and VNN-infected groups and each tissue separately using Hmisc version 4.7–0 package in R v4.1.1 [71]. Subsequently, based on the differential gene expression and correlation analysis results, GCTA v1.94.1 LOCO (–mlma-loco) was used to identify expression QTL for the candidate VNN resistance genes in each tissue and condition separately.

### Functional characterization of the VNN resistance QTL region in the European sea bass genome

Multiple functional genomics datasets (including RNA-seq, ATAC-seq and ChIP-seq) generated from naïve juvenile seabass treated with Polyinosinic:polycytidylic acid [poly(I:C)], a synthetic immunostimulant used to simulate double-stranded RNA (dsRNA) viral infections, were used to functionally annotate the VNN resistance QTL region in the European sea bass genome. Briefly, individuals were stimulated by intraperitoneal injection with PBS or Poly(I:C) (6 replicates/condition). Animals were sacrificed 24 h post-infection (hpi) and head kidney sampled for RNA-seq, ATAC-seq and ChIP-seq library preparation (6 replicates/condition). RNA-seq, ATAC-seq and ChIP-seq libraries were also produced for brain of healthy juvenile sea bass (3 males and 3 females). All these procedures were part of a large annotation effort under the framework of the EU project AQUA-FAANG (https://www.aqua-faang.eu/).

For all the abovementioned samples, total RNA was extracted using RNeasy Mini Kit (Qiagen, Hilden, Germany) according to the instructions of the manufacturer, and high-quality RNA was then sent to Novogene for cDNA library preparation and sequencing on the Illumina NovaSeq 6000 system platform (Illumina, California, San Diego, USA) as 150 bp paired-end reads.

From the same samples, ATAC-seq libraries were constructed using the Omni-ATAC protocol [72] with species and tissue-specific modifications (details here: https://data.faang.org/api/fire_api/experiments/NMBU_SOP_OmniATAC_protocol_20200429.pdf). Briefly, frozen tissue fragments (20–30 mg) were homogenized by using a pre-chilled 2-ml Dounce tissue homogenizer containing homogenization buffer. Connective tissue and residual debris were pre-cleaned by filtration through a 70-μm nylon filter. Intact nuclei were then collected by density gradient centrifugation over iodixanol (25% iodixanol, 29% iodixanol, 35% iodixanol). For brain samples, after PBS washing, nuclei were counted by trypan blue staining and 50,000 nuclei were then recruited for Tn5 transposase reaction by using Illumina Tagment DNA Enzyme and Buffer kit (Illumina, California, San Diego, USA). For head kidney samples, all washing steps were skipped in order to avoid nuclei aggregation and 1 µl of nuclei band was directly employed for transposase reaction. Transposition reactions were cleaned up with MinElute PCR Purification Kit (Qiagen, Hilden, Germany) and uniquely barcoded libraries were obtained following amplification using Illumina Nextera DNA Unique Dual Indexes (Illumina, California, San Diego, USA). After quality assessment, all libraries were sent to Novogene (Cambridge, UK) for 150 bp paired-end sequencing on the Illumina NovaSeq 6000 system platform. Sequence data analyses were performed using the NF-core pipeline for ATAC-seq analysis (nf-core/atacseq v1.2.1, https://github.com/nf-core/atacseq).

Following the manufacturer’s instructions, ChIP-seq libraries for the same samples were prepared using the µChIPmentation Kit for Histones (Diagenode). Three histone marks were investigated: H3K4me3 (promoter regions), H3K27ac (active enhancer and promoter regions) and H3K27me3 (associated with Polycomb repression complexes). After quality assessment, all libraries were sent to Novogene and sequenced by Illumina NovaSeq6000 150PE in order to obtain 45 M reads per sample. All sequencing data analyses were conducted through the NF-core ChIP-seq analysis pipeline (https://github.com/nf-core/chipseq).

Additionally, a total of 20 fish (10 mock- and 10 VNN-infected) from the transcriptome immune challenge experiment were used to assess chromatin accessibility in the brain and head kidney tissue under mock and VNN infection conditions. Forty ATAC-seq libraries were constructed from frozen tissue using the procedures described above, and sequenced by Novogene (Cambridge, UK) as 150 bp paired-end reads using the Illumina NovaSeq 6000 platform. This data was also analysed using the NF-core pipeline for ATAC-seq analysis (nf-core/atacseq v1.2.1, https://github.com/nf-core/atacseq).

For each sample and condition (i.e. PBS-stimulated HKs, Poly(I:C)-stimulated HKs, brains from male and female individuals), ATAC-seq and ChIP-seq data were combined to infer genome-wide chromatin state and associated regulatory elements using ChromHMM v1.24 [73], assuming 10 different chromatin states. This information was crossed with genetic variation in the VNN resistance QTL region, and the putative impact of variants overlapping regulatory elements on putative transcription factor binding sites was predicted using a web-based software FABIAN-variant v2022-05–26 [74]. Briefly, 41-bp sequence fragments centred around each variant were analysed on the web-based platform of the software, with the transcription factor flexible models (TFFMs)-detailed model option.

### Comparative genomic sequence analysis

The Interferon Alpha Inducible Protein 27-like genes (*IFI27L2A* and *IFI27L2*) encoding protein sequences in the European sea bass genome (GCF_905237075.1), the striped bass (*Morone saxatilis*) genome (GCF_004916995.1) and the gilthead sea bream (*Sparus aurata*) genome (CA_900880675.1) were aligned using MUSCLE [75]. Striped bass is phylogenetically very close to European sea bass (same family, Moronidae), while gilthead sea bream is phylogenetically more distant. From European sea bass, sequence XM_051391909.1 (encoded by locus LOC127358638) was too divergent to be reliably aligned with the other IFI27L2/2A transcripts and hence was removed from the analysis. Multiple alignment of the remaining sequences was cleaned using Noisy v1.5.12 [76]. The final alignment was used as input for reconstructing a gene tree using maximum likelihood (PhyML + SMS) and Bayesian methods (MrBayes). To assess the genomic evolution conservation of the *IFI27L2/2A* genes between European sea bass and striped bass multiple sequence alignment of the genomic regions harbouring these genes in two species (European sea bass: Chromosome 3: 10,075168–10,097,914; Striped sea bass: Chromosome X: 15,596,712–15,606,764) was performed using mVISTA [77]. Additionally, to assess the gene evolutionary similarity between the five copies of the IFI27L2/2A genes in European sea bass multiple sequence alignment of the protein sequences of the five IFI27L2/2A gene loci in the VNN QTL region was performed using MEGA11 v0.10 software [78] and visualized using a Bioconductor R package ggmsa v1.3.4 [79]

## Supplementary Information

Additional file 1: Fig. S1 Barplot showing offspring contribution to the study population by the parents used in the current study, A) Sires and B) Dams. Table S1 Wild populations allele frequencies of four variants associated with VNN resistance located in the 3’UTR region of the ENSDLAG00005026832gene. Fig. S2 Predicted TFBS loss or gain effect results summary from FABIAN-variant of two putative causal variants

Additional file 2: Table S1 Genome-wide associations analysis results and annotations for variants significantly associated with VNN resistance defined as binary survivalat the end of the VNN challenge experiment. Table S2 Genome-wide associations analysis results and annotations for variants significantly associated with VNN resistance defined as days to death during the VNN challenge experiment

## Data Availability

Genotypes and phenotypes have been submitted to the Europeans Variant Archive (EVA). Whole-genome raw sequencing data have been uploaded to the NCBI Short Read Archive (SRA) under BioProject accession PRJNA1110973 (https://www.ncbi.nlm.nih.gov/bioproject/PRJNA1110973/). RNA-sequencing data have also been uploaded to the NCBI SRA under BioProject accession PRJNA1122486 (https://www.ncbi.nlm.nih.gov/bioproject/PRJNA1122486/). ATAC-seq and ChIP-seq sequencing data, and functional annotation from head kidney samples have been uploaded to the EMBL-EBI repository under accessions PRJEB52284 (https://www.ncbi.nlm.nih.gov/bioproject/PRJEB52284/) and PRJEB59557 (https://www.ncbi.nlm.nih.gov/bioproject/PRJEB59557/), respectively. ATAC-seq and ChIP-seq sequencing data from brain samples have been uploaded to the EMBL-EBI repository under accessions PRJEB52775 (https://www.ncbi.nlm.nih.gov/bioproject/PRJEB52775/) and PRJEB59432 (https://www.ncbi.nlm.nih.gov/bioproject/PRJEB59432/), respectively.

## Abbreviations

VNN: Viral nervous necrosis
NNV: Nervos necrosis virus
QTL: Quantitative trait locus
LG: Linkage group
Mb: Megabase
ATAC-seq: Assay for transposase-accessible chromatin sequencing
ChIP-seq: Chromatin immunoprecipitation sequencing
SNP: Single-nucleotide polymorphism
DD: Days to death
BS: Binary survival
GWAS: Genome-wide association study
WGS: Whole-genome sequencing
NCBI: National Center for Biotechnology Information
RNA-seq: RNA sequencing
TSS: Transcription start site
TTS: Transcription termination site
RR VNN: QTL resistance genotype
RS VNN: QTL heterozygoty genotype
SS VNN: QTL susceptible genotype
CHR: Chromosome
Hk: Head kidney
Br: Brain
ISG: Interferon inducible gene
miRNA: MicroRNA
RBP: RNA-binding protein
DMEM: Dulbecco’s modified Eagle’s medium
QC: Quality control
HWE: Hardy-Weinberg equilibrium
BY: Benjamini-Yekutieli
GEBV: Genomic estimated breeding value
GBLUP: Genomic best linear unbiased prediction
TFFM: Transcription factor flexible models
FDR: False discovery rate
FC: Fold change

## References

[1] Vandeputte M, Gagnaire PA, Allal F. The European sea bass: a key marine fish model in the wild and in aquaculture. Anim Genet. 2019. 10.1111/age.12779. 30883830 10.1111/age.12779 PMC6593706

[2] FAO, *Dicentrarchus labrax Linnaeus,*1758*.* In: Fisheries and Aquaculture*.* The Food and Agriculture Organization of the United Nations. 2024. https://www.fao.org/fishery/en/aqspecies/2291/en. Accessed 30 May 2024.

[3] Muniesa A, Basurco B, Aguilera C, Furones D, Reverté C, Sanjuan-Vilaplana A, Jansen MD, Brun E, Tavornpanich S. Mapping the knowledge of the main diseases affecting sea bass and sea bream in Mediterranean. Transbound Emerg Dis. 2020. 10.1111/tbed.13482. 31960605 10.1111/tbed.13482

[4] Barsøe S, Allal F, Vergnet A, Vandeputte M, Olesen NJ, Schmidt JG, Larsen CA, Cuenca A, Vendramin N. Different survival of three populations of European sea bass (Dicentrarchus labrax) following challenge with two variants of nervous necrosis virus (NNV). Aquac Rep. 2021. 10.1016/j.aqrep.2021.100621.

[5] Le Breton A, Grisez L, Sweetman J, Ollevier F. Viral nervous necrosis (VNN) associated with mass mortalities in cage‐reared sea bass, Dicentrarchus labrax (L.). J Fish Dis. 1997. 10.1046/j.1365-2761.1997.00284.x.

[6] Chi SC, Wu YC, Hong JR. Nodaviruses of fish. In: Kibenge FSB, Godoy MG, editors. Aquaculture virology. San Diego: Academic Press; 2016. p. 371–93.

[7] Yang Z, Yue GH, Wong SM. VNN disease and status of breeding for resistance to NNV in aquaculture. Aquac Fish. 2022. 10.1016/j.aaf.2021.04.001.

[8] Doan KQ, Vandeputte M, Chatain B, Haffray P, Vergnet A, Breuil G, Allal F. Genetic variation of resistance to Viral Nervous Necrosis and genetic correlations with production traits in wild populations of the European sea bass (Dicentrarchus labrax). Aquac. 2017. 10.1016/j.aquaculture.2017.05.0118.

[9] Griot R, Allal F, Phocas F, Brard-Fudulea S, Morvezen R, Bestin A, Haffray P, François Y, Morin T, Poncet C, Vergnet A. Genome-wide association studies for resistance to viral nervous necrosis in three populations of European sea bass (Dicentrarchus labrax) using a novel 57k SNP array DlabChip. Aquac. 2021. 10.1016/j.aquaculture.2020.735930.

[10] Faggion S, Bertotto D, Babbucci M, Dalla Rovere G, Franch R, Bovolenta M, Laureau S, Pascoli F, Toffan A, Bargelloni L, Carnier P. Resistance to viral nervous necrosis in European sea bass (Dicentrarchus labrax L.): heritability and relationships with body weight, cortisol concentration, and antibody titer. Genet Sel Evol. 2021. 10.1186/s12711-021-00625-2. 10.1186/s12711-021-00625-2 PMC8017662 33794770

[11] Palaiokostas C, Cariou S, Bestin A, Bruant JS, Haffray P, Morin T, Cabon J, Allal F, Vandeputte M, Houston RD. Genome-wide association and genomic prediction of resistance to viral nervous necrosis in European sea bass (Dicentrarchus labrax) using RAD sequencing. Genet Sel Evol. 2018. 10.1186/s12711-018-0401-2. 29884113 10.1186/s12711-018-0401-2 PMC5994081

[12] Vela-Avitúa S, Thorland I, Bakopoulos V, Papanna K, Dimitroglou A, Kottaras E, Leonidas P, Guinand B, Tsigenopoulos CS, Aslam ML. Genetic basis for resistance against viral nervous necrosis: GWAS and potential of genomic prediction explored in farmed European sea bass (Dicentrarchus labrax). Front Genet. 2022. 10.3389/fgene.2022.804584. 35401661 10.3389/fgene.2022.804584 PMC8992836

[13] Vandeputte M, Chatain B, Haffray P, Vergnet A, Breuil G, Allal F. Genetic variation of resistance to Viral Nervous Necrosis and genetic correlations with production traits in wild populations of the European sea bass (Dicentrarchus labrax). Aquac. 2017. 10.1016/j.aquaculture.2017.05.011.

[14] Delpuech E, Vandeputte M, Morvezen R, Bestin A, Besson M, Brunier J, Bajek A, Imarazene B, François Y, Bouchez O, Cousin X. Whole-genome sequencing identifies interferon-induced protein IFI6/IFI27-like as a strong candidate gene for VNN resistance in European sea bass. Genet Sel Evol. 2023. 10.1186/s12711-023-00805-2. 37143017 10.1186/s12711-023-00805-2 PMC10161657

[15] Tine M, Kuhl H, Gagnaire PA, Louro B, Desmarais E, Martins RS, Hecht J, Knaust F, Belkhir K, Klages S, Dieterich R. European sea bass genome and its variation provide insights into adaptation to euryhalinity and speciation. Nat Commun. 2014. 10.1038/ncomms6770. 25534655 10.1038/ncomms6770 PMC4284805

[16] Clark EL, Archibald AL, Daetwyler HD, Groenen MA, Harrison PW, Houston RD, Kühn C, Lien S, Macqueen DJ, Reecy JM, Robledo D. From FAANG to fork: application of highly annotated genomes to improve farmed animal production. Genome Biol. 2020. 10.1186/s13059-020-02197-8. 33234160 10.1186/s13059-020-02197-8 PMC7686664

[17] Martínez P, Robledo D, Taboada X, Blanco A, Moser M, Maroso F, Hermida M, Gómez-Tato A, Álvarez-Blázquez B, Cabaleiro S, Piferrer F. A genome-wide association study, supported by a new chromosome-level genome assembly, suggests sox2 as a main driver of the undifferentiatiated ZZ/ZW sex determination of turbot (Scophthalmus maximus). Genom. 2021. 10.1016/j.ygeno.2021.04.007. 10.1016/j.ygeno.2021.04.007 33838278

[18] de la Herrán R, Hermida M, Rubiolo JA, Gómez-Garrido J, Cruz F, Robles F, Navajas-Pérez R, Blanco A, Villamayor PR, Torres D, Sánchez-Quinteiro P. A chromosome-level genome assembly enables the identification of the follicule stimulating hormone receptor as the master sex-determining gene in the flatfish Solea senegalensis. Mol Ecol Resour. 2023. 10.1111/1755-0998.13750. 36587276 10.1111/1755-0998.13750

[19] Etherington GJ, Nash W, Ciezarek A, Mehta TK, Barria A, Penaloza C, Khan MG, Durrant A, Forrester N, Fraser F, Irish N. Chromosome-level genome sequence of the Genetically Improved Farmed Tilapia (GIFT, Oreochromis niloticus) highlights regions of introgression with O. mossambicus. BMC genomics. 2022. 10.1186/s12864-022-09065-8. 10.1186/s12864-022-09065-8 PMC9756657 36522771

[20] Emilsson V, Thorleifsson G, Zhang B, Leonardson AS, Zink F, Zhu J, Carlson S, Helgason A, Walters GB, Gunnarsdottir S, Mouy M. Genetics of gene expression and its effect on disease. Nat. 2008. 10.1038/nature06758. 10.1038/nature06758 18344981

[21] Albert FW, Kruglyak L. The role of regulatory variation in complex traits and disease. Nat Rev Genet. 2015. 10.1038/nrg3891. 25707927 10.1038/nrg3891

[22] GTEx Consortium. The GTEx Consortium atlas of genetic regulatory effects across human tissues. Science. 2020. 10.1126/science.aaz1776. 10.1126/science.aaz1776 PMC7737656 32913098

[23] Liu S, Gao Y, Canela-Xandri O, Wang S, Yu Y, Cai W, Li B, Xiang R, Chamberlain AJ, Pairo-Castineira E, D’Mellow K. A multi-tissue atlas of regulatory variants in cattle. Nat Genet. 2022. 10.1038/s41588-022-01153-5. 35953587 10.1038/s41588-022-01153-5 PMC7613894

[24] Teng J, Gao Y, Yin H, Bai Z, Liu S, Zeng H, PigGTEx Consortium, Bai L, Cai Z, Zhao B, Li X. A compendium of genetic regulatory effects across pig tissues. Nat Genet. 2024. 10.1038/s41588-023-01585-7. 10.1038/s41588-023-01585-7 PMC10786720 38177344

[25] Buenrostro JD, Wu B, Chang HY, Greenleaf WJ. ATAC-seq: a method for assaying chromatin accessibility genome-wide. Curr Protoc Mol Biol. 2015. 10.1002/0471142727.mb2129s109. 25559105 10.1002/0471142727.mb2129s109 PMC4374986

[26] Zhang Y, Liu T, Meyer CA, Eeckhoute J, Johnson DS, Bernstein BE, Nusbaum C, Myers RM, Brown M, Li W, Liu XS. Model-based analysis of ChIP-Seq (MACS). Genome Biol. 2008. 10.1186/gb-2008-9-9-r137. 18798982 10.1186/gb-2008-9-9-r137 PMC2592715

[27] Faggion S, Bertotto D, Bonfatti V, Freguglia M, Bargelloni L, Carnier P. Genomic predictions of phenotypes and pseudo-phenotypes for viral nervous necrosis resistance, cortisol concentration, antibody titer and body weight in european sea bass. Animals. 2022. 10.3390/ani12030367. 35158690 10.3390/ani12030367 PMC8833701

[28] Mukiibi R, Robledo D, Peñaloza C, Ferraresso S, Franch R, Bertotto D, Freguglia M, Laureau S, Pascoli F, Toffan A, Tsigenopolous C. A major QTL affects resistance to viral nervous necrosis in farmed European seabass. In: Veerkamp RF, de Haas Y, editors, Proceedings of 12th World Congress on Genetics Applied to Livestock Production (WCGALP) Technical and species orientated innovations in animal breeding, and contribution of genetics to solving societal challenges, Wageningen: Wageningen Academic Publishers; 2022. p. 2375–2378. 10.3920/978-90-8686-940-4.

[29] Peñaloza C, Manousaki T, Franch R, Tsakogiannis A, Sonesson AK, Aslam ML, Allal F, Bargelloni L, Houston RD, Tsigenopoulos CS. European seabass and gilthead seabream population pools used for SNP discovery for the development of the 60K combined-species MedFish SNP array. SRA: PRJEB40423. 2020. https://www.ncbi.nlm.nih.gov/bioproject/PRJEB40423/.

[30] Smith NC, Rise ML, Christian SL. A comparison of the innate and adaptive immune systems in cartilaginous fish, ray-finned fish, and lobe-finned fish. Front Immunol. 2019. 10.3389/fimmu. 31649660 10.3389/fimmu.2019.02292 PMC6795676

[31] Mokhtar DM, Zaccone G, Alesci A, Kuciel M, Hussein MT, Sayed RK. Main components of fish immunity: An overview of the fish immune system. Fishes. 2023. 10.3390/fishes8020093.

[32] Mancuso N, Freund MK, Johnson R, Shi H, Kichaev G, Gusev A, Pasaniuc B. Probabilistic fine-mapping of transcriptome-wide association studies. Nat Genet. 2019. 10.1038/s41588-019-0367-1. 30926970 10.1038/s41588-019-0367-1 PMC6619422

[33] Schoggins JW. Recent advances in antiviral interferon-stimulated gene biology. F1000Research. 2018. 10.12688/f1000research.12450.1. 10.12688/f1000research.12450.1 PMC5850085 29568506

[34] Lucas TM, Richner JM, Diamond MS. The interferon-stimulated gene Ifi27l2a restricts West Nile virus infection and pathogenesis in a cell-type-and region-specific manner. J Virol. 2016. 10.1128/jvi.02463-15. 26699642 10.1128/JVI.02463-15 PMC4810731

[35] Guo J, Huang W, Zhao X, Ji N, Chen K, Shi Y, Feng J, Zou J, Wang J. The expanded ISG12 family in zebrafish: ISG12. 1 suppresses virus replication via targeting viral phosphoprotein. Dev Comp Immunol. 2023. 10.1016/j.dci.2023.104672. 10.1016/j.dci.2023.104672 36822549

[36] Nuñez-Ortiz N, Pascoli F, Picchietti S, Buonocore F, Bernini C, Toson M, Scapigliati G, Toffan A. A formalin-inactivated immunogen against viral encephalopathy and retinopathy (VER) disease in European sea bass (Dicentrarchus labrax): Immunological and protection effects. Vet Res. 2016. 10.1186/s13567-016-0376-3. 27590537 10.1186/s13567-016-0376-3 PMC5010674

[37] Toubanaki DK, Efstathiou A, Karagouni E. Transcriptomic analysis of fish hosts responses to nervous necrosis virus. Pathogens. 2022. 10.3390/pathogens11020201. 35215144 10.3390/pathogens11020201 PMC8875540

[38] Lynch M, Force A. The probability of duplicate gene preservation by subfunctionalization. Genetics. 2000. 10.1093/genetics/154.1.459. 10629003 10.1093/genetics/154.1.459 PMC1460895

[39] Kim S, Wysocka J. Deciphering the multi-scale, quantitative cis-regulatory code. Mol Cell. 2023. 10.1016/j.molcel.2022.12.032. 36693380 10.1016/j.molcel.2022.12.032 PMC9898153

[40] Carter B, Zhao K. The epigenetic basis of cellular heterogeneity. Nat Rev Genet. 2021. 10.1038/s41576-020-00300-0. 33244170 10.1038/s41576-020-00300-0 PMC10880028

[41] Wang Q, Peng C, Yang M, Huang F, Duan X, Wang S, Cheng H, Yang H, Zhao H, Qin Q. Single-cell RNA-seq landscape midbrain cell responses to red spotted grouper nervous necrosis virus infection. PLoS Pathog. 2021. 10.1371/journal.ppat.1009665. 34185811 10.1371/journal.ppat.1009665 PMC8241073

[42] El-Mayet FS, Sawant L, Thunuguntla P, Jones C. Combinatorial effects of the glucocorticoid receptor and Krüppel-like transcription factor 15 on bovine herpesvirus 1 transcription and productive infection. J Virol. 2017. 10.1128/jvi.00904-17. 28794031 10.1128/JVI.00904-17 PMC5640833

[43] Luo X, Zhang R, Lu M, Liu S, Baba HA, Gerken G, Wedemeyer H, Broering R. Hippo pathway counter-regulates innate immunity in hepatitis B virus infection. Front immunol. 2021. 10.3389/fimmu.2021.684424. 34113355 10.3389/fimmu.2021.684424 PMC8185339

[44] Carrizo V, Valenzuela CA, Zuloaga R, Aros C, Altamirano C, Valdés JA, Molina A. Effect of cortisol on the immune-like response of rainbow trout (Oncorhynchus mykiss) myotubes challenged with Piscirickettsia salmonis. Vet Immunol Immunopathol. 2021. 10.1016/j.vetimm.2021.110240. 33962313 10.1016/j.vetimm.2021.110240

[45] Lama R, Pereiro P, Valenzuela-Muñoz V, Gallardo-Escárate C, Tort L, Figueras A, Novoa B. RNA-Seq analysis of European sea bass (Dicentrarchus labrax L.) infected with nodavirus reveals powerful modulation of the stress response. Vet Res. 2020. 10.1186/s13567-020-00784-y. 10.1186/s13567-020-00784-y PMC7218500 32398117

[46] Andersson R, Sandelin A. Determinants of enhancer and promoter activities of regulatory elements. Nat Rev Genet. 2020. 10.1038/s41576-019-0173-8. 31605096 10.1038/s41576-019-0173-8

[47] Malfait J, Wan J, Spicuglia S. Epromoters are new players in the regulatory landscape with potential pleiotropic roles. BioEssays. 2023. 10.1002/bies.202300012. 37246247 10.1002/bies.202300012

[48] Abell NS, DeGorter MK, Gloudemans MJ, Greenwald E, Smith KS, He Z, Montgomery SB. Multiple causal variants underlie genetic associations in humans. Science. 2022. 10.1126/science.abj5117. 10.1126/science.abj5117 PMC9725108 35298243

[49] Mayya VK, Duchaine TF. Ciphers and executioners: how 3′-untranslated regions determine the fate of messenger RNAs. Front Genet. 2019. 10.3389/fgene.2019.00006. 30740123 10.3389/fgene.2019.00006 PMC6357968

[50] Fauvel C, Boryshpolets S, Cosson J, Wilson Leedy JG, Labbé C, Haffray P, Suquet M. Improvement of chilled seabass sperm conservation using a cell culture medium. J Appl Ichthyol. 2012. 10.1111/jai.12071.

[51] Panzarin V, Fusaro A, Monne I, Cappellozza E, Patarnello P, Bovo G, Capua I, Holmes EC, Cattoli G. Molecular epidemiology and evolutionary dynamics of betanodavirus in southern Europe. Infection GenetiEvolu. 2012. 10.1016/j.meegid.2011.10.007. 10.1016/j.meegid.2011.10.007 22036789

[52] Walsh PS, Metzger DA, Higuchi R. Chelex 100 as a medium for simple extraction of DNA for PCR-based typing from forensic material. Biotechniques. 1991. 10.2144/000114018. 1867860

[53] Peñaloza C, Manousaki T, Franch R, Tsakogiannis A, Sonesson AK, Aslam ML, Allal F, Bargelloni L, Houston RD, Tsigenopoulos CS. Development and testing of a combined species SNP array for the European seabass (Dicentrarchus labrax) and gilthead seabream (Sparus aurata). Genomics. 2021. 10.1016/j.ygeno.2021.04.038. 33933591 10.1016/j.ygeno.2021.04.038 PMC8276775

[54] Aljanabi SM, Martinez I. Universal and rapid salt-extraction of high quality genomic DNA for PCR-based techniques. Nucleic Acids Res. 1997. 10.1093/nar/25.22.4692. 9358185 10.1093/nar/25.22.4692 PMC147078

[55] Andrews S. FastQC: a quality control tool for high throughput sequence data. 2010. https://www.bioinformatics.babraham.ac.uk/projects/fastqc/. Accessed 19 Jan 2023.

[56] Bolger AM, Lohse M, Usadel B. Trimmomatic: a flexible trimmer for Illumina sequence data. Bioinformatics. 2014. 10.1093/bioinformatics/btu170. 24695404 10.1093/bioinformatics/btu170 PMC4103590

[57] Li H, Durbin R. Fast and accurate short read alignment with Burrows-Wheeler transform. Bioinformatics. 2009. 10.1093/bioinformatics/btp324. 19451168 10.1093/bioinformatics/btp324 PMC2705234

[58] Li H. A statistical framework for SNP calling, mutation discovery, association mapping and population genetical parameter estimation from sequencing data. Bioinformatics. 2011. 10.1093/bioinformatics/btr509. 21903627 10.1093/bioinformatics/btr509 PMC3198575

[59] Chang CC, Chow CC, Tellier LC, Vattikuti S, Purcell SM, Lee JJ. Second-generation PLINK: rising to the challenge of larger and richer datasets. Gigascience. 2015. 10.1186/s13742-015-0047-8. 25722852 10.1186/s13742-015-0047-8 PMC4342193

[60] Griot R, Allal F, Brard-Fudulea S, Morvezen R, Haffray P, Phocas F, Vandeputte M. APIS: An auto-adaptive parentage inference software that tolerates missing parents. Mol Ecol Resour. 2020. 10.1111/1755-0998.13103. 31609085 10.1111/1755-0998.13103

[61] Danecek P, Auton A, Abecasis G, Albers CA, Banks E, DePristo MA, Handsaker RE, Lunter G, Marth GT, Sherry ST, McVean G. The variant call format and VCFtools. Bioinformatics. 2011. 10.1093/bioinformatics/btr330. 21653522 10.1093/bioinformatics/btr330 PMC3137218

[62] Sargolzaei M, Chesnais JP, Schenkel FS. A new approach for efficient genotype imputation using information from relatives. BMC Genomics. 2014. 10.1186/1471-2164-15-478. 24935670 10.1186/1471-2164-15-478 PMC4076979

[63] Kassambara A, Kosinski M, Biecek P. *survminer R package: Survival Data Analysis and Visualization.* 2016. https://cran.r-project.org/web/packages/survminer/survminer.pdf. Accessed 19 Jan 2024.

[64] Yang J, Lee SH, Goddard ME, Visscher PM. GCTA: a tool for genome-wide complex trait analysis. Am J Hum Genet. 2011. 10.1016/j.ajhg.2010.11.011. 21167468 10.1016/j.ajhg.2010.11.011 PMC3014363

[65] Benjamini Y, Yekutieli D. The control of the false discovery rate in multiple testing under dependency. Ann Stat. 2001;29(4):1165–88.

[66] Bushnell B. BBTools software package*.* 2014;sourceforge.net/projects/bbmap/.

[67] Dobin A, Davis CA, Schlesinger F, Drenkow J, Zaleski C, Jha S, Batut P, Chaisson M, Gingeras TR. STAR: ultrafast universal RNA-seq aligner. Bioinformatics. 2013. 10.1093/bioinformatics/bts635. 23104886 10.1093/bioinformatics/bts635 PMC3530905

[68] Liao Y, Smyth GK, Shi W. featureCounts: an efficient general purpose program for assigning sequence reads to genomic features. Bioinformatics. 2014. 10.1093/bioinformatics/btt656. 24227677 10.1093/bioinformatics/btt656

[69] Love MI, Huber W, Anders S. Moderated estimation of fold change and dispersion for RNA-seq data with DESeq2. Genome Biol. 2014. 10.1186/s13059-014-0550-8. 25516281 10.1186/s13059-014-0550-8 PMC4302049

[70] Peruzza L, Gerdol M, Oliphant A, Wilcockson D, Pallavicini A, Hawkins L, Thatje S, Hauton C. The consequences of daily cyclic hypoxia on a European grass shrimp: From short-term responses to long-term effects. Funct Ecol. 2018. 10.1111/1365-2435.13150.

[71] Harrell Jr FE, Harrell Jr MF. Package ‘hmisc’. CRAN2018. 2019;235–236.

[72] Corces MR, Trevino AE, Hamilton EG, Greenside PG, Sinnott-Armstrong NA, Vesuna S, Satpathy AT, Rubin AJ, Montine KS, Wu B, Kathiria A. An improved ATAC-seq protocol reduces background and enables interrogation of frozen tissues. Nat Methods. 2017. 10.1038/nmeth.4396. 28846090 10.1038/nmeth.4396 PMC5623106

[73] Ernst J, Kellis M. Chromatin-state discovery and genome annotation with ChromHMM. Nat Protoc. 2017. 10.1038/nprot.2017.124. 29120462 10.1038/nprot.2017.124 PMC5945550

[74] Steinhaus R, Robinson PN, Seelow D. FABIAN-variant: predicting the effects of DNA variants on transcription factor binding. Nucleic Acids Res. 2022. 10.1093/nar/gkac393. 35639768 10.1093/nar/gkac393 PMC9252790

[75] Edgar RC. MUSCLE: multiple sequence alignment with high accuracy and high throughput. Nucleic Acids Res. 2004. 10.1093/nar/gkh340. 15034147 10.1093/nar/gkh340 PMC390337

[76] Dress AW, Flamm C, Fritzsch G, Grünewald S, Kruspe M, Prohaska SJ, Stadler PF. Noisy: identification of problematic columns in multiple sequence alignments. Algorithms Mol Biol. 2008. 10.1186/1748-7188-3-7. 18577231 10.1186/1748-7188-3-7 PMC2464588

[77] Frazer KA, Pachter L, Poliakov A, Rubin EM, Dubchak I. VISTA: computational tools for comparative genomics. Nucleic Acids Res. 2004. 10.1093/nar/gkh458. 15215394 10.1093/nar/gkh458 PMC441596

[78] Tamura K, Stecher G, Kumar S. MEGA11: molecular evolutionary genetics analysis version 11. Mol Biol Evol. 2021. 10.1093/molbev/msab120. 33892491 10.1093/molbev/msab120 PMC8233496

[79] Zhou L, Feng T, Xu S, Gao F, Lam TT, Wang Q, Wu T, Huang H, Zhan L, Li L, Guan Y. ggmsa: a visual exploration tool for multiple sequence alignment and associated data. Brief Bioinform. 2022. 10.1093/bib/bbac222. 35671504 10.1093/bib/bbac222

