# Supplementary material for: Integrated functional genomic analysis identifies regulatory variants underlying a major QTL for disease resistance in European sea bass
Source: BMC Biol. 2025 Mar 11;23:75. doi: 10.1186/s12915-025-02180-4 (PMC11899128; doi:10.1186/s12915-025-02180-4)
Supplement: Supplementary file 1 — Additional file 1: Fig. S1 Barplot showing offspring contribution to the study population by the parents used in the current study, A) Sires and B) Dams. Table S1 Wild populations allele frequencies of four variants associated with VNN resistance located in the 3’UTR region of the ENSDLAG00005026832gene. Fig. S2 Predicted TFBS loss or gain effect results summary from FABIAN-variant of two putative causal variants [file 12915_2025_2180_MOESM1_ESM.pdf]

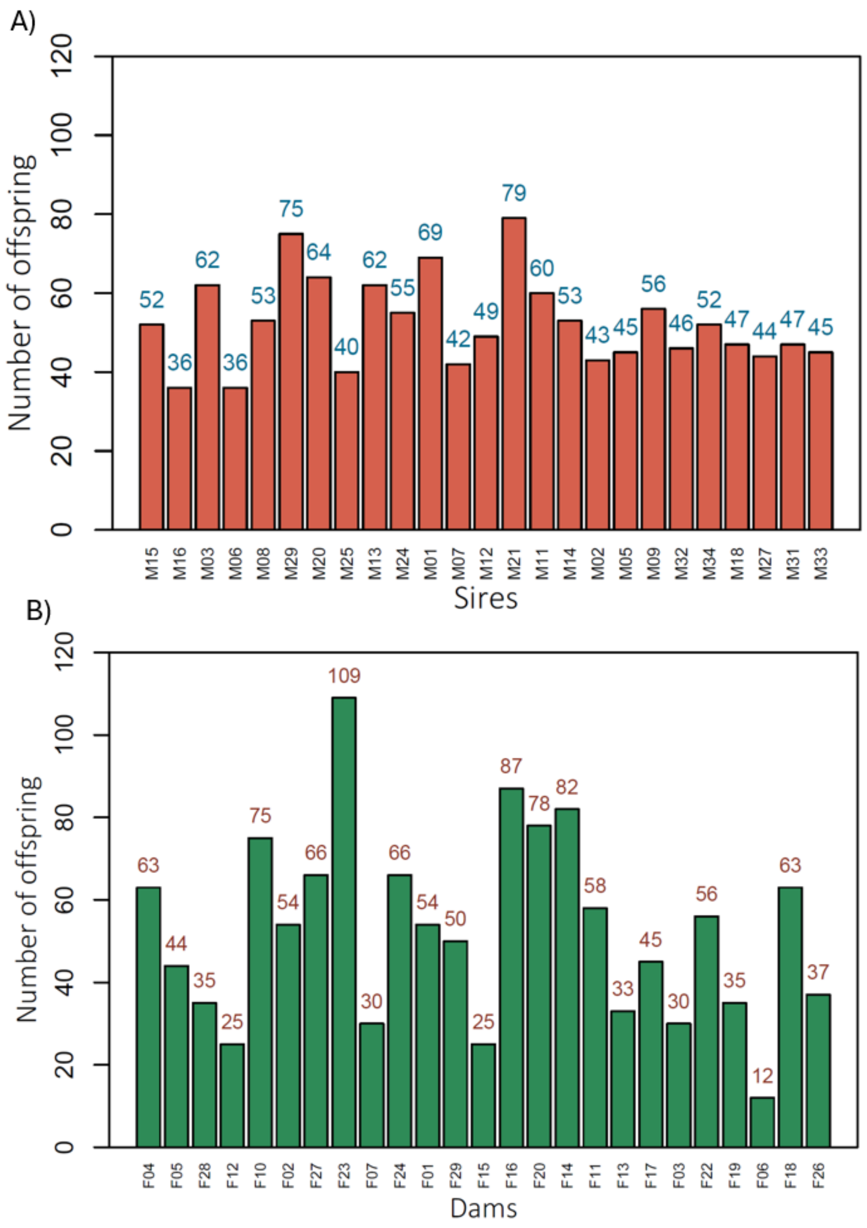

Figure S1: Barplot showing offspring contribution to the study population by the parents used in the current study, A) Sires and B) Dams.

Table S1: Wild populations allelic frequencies of 4 of the variants associated with VNN resistance located in the 3'UTR region of the *ENSDLAG00005026832 (IFI27L2A)* gene.

	Allele		*ATL*		*WMED*		*EMED*	
Variant	*Reference (Ref)*	*Alternative (Alt)*	*Ref*	*Alt*	*Ref*	*Alt*	*Ref*	*Alt*
CHR3:10081901	T	G	0.85	0.15	0.87	0.13	0.25	0.75
CHR3:10081923	C	T	0.92	0.08	0.95	0.05	0.27	0.73
CHR3:10082148	C	T	0.77	0.23	0.75	0.25	0.18	0.82
CHR3:10082380	T	C	0.65	0.35	0.80	0.20	0.18	0.82

Alternative allele – VNN resistance allele, Reference allele – susceptible allele, ATL – Atlantic population (susceptible), WMED – West Mediterranean population (susceptible), EMED – East Mediterranean population (resistant).

VARIANT: CHR3:10077301

CCTGGTGAGTACCACTGTTACTTTGACAATT>CCTGGTGAGTACCACTATTACTTTGACAATT

TFBS	ModelID	Database	WT score	MT score	WT strand	MT strand	Prediction	Score
KLF15	TFFM0942.1	jaspar2022DetailedTFFMs	0.0225	0.6857	minus	plus	gain	0.8039

***TFBS**: Name of the transcription factor, **ModelID**: ID of the model in the source database, **Database**: Source database of the model, **wt_score, mt_score**: The highest score in the reference and mutated sequence, **wt_strand, mt_strand**: Strand of the location with the highest score in the reference and mutated sequence, **Prediction**: Prediction of a gain or loss of TFBS, or NA if not prediction was possible, **Score**: Score of prediction between -1 (likely TFBS loss) and +1 (likely TFBS gain)

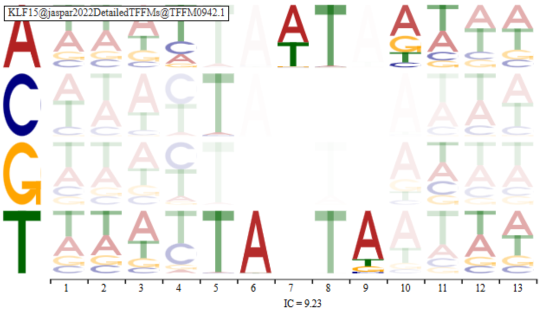

VARIANT: CHR3:10081209

CTGCAGGTTTCACCTCAGCTGGAGTAGCTGCAGGCTCCATT>CTGCAGGTTTCACCTCAGCTGGAATAGCTGCAGGCTCCATT

TFBS	ModelID	Database	WT score	MT score	WT strand	MT strand	Prediction	Score
TEAD4	TFFM0110.1	jaspar2022DetailedTFFMs	0.0201	0.7912	minus	plus	gain	0.9392
ZNF140	TFFM0634.1	jaspar2022DetailedTFFMs	0.0132	0.8483	plus	plus	gain	0.9808

***TFBS**: Name of the transcription factor, **ModelID**: ID of the model in the source database, **Database**: Source database of the model, **wt_score, mt_score**: The highest score in the reference and mutated sequence, **wt_strand, mt_strand**: Strand of the location with the highest score in the reference and mutated sequence, **Prediction**: Prediction of a gain or loss of TFBS, or NA if not prediction was possible, **Score**: Score of prediction between -1 (likely TFBS loss) and +1 (likely TFBS gain)

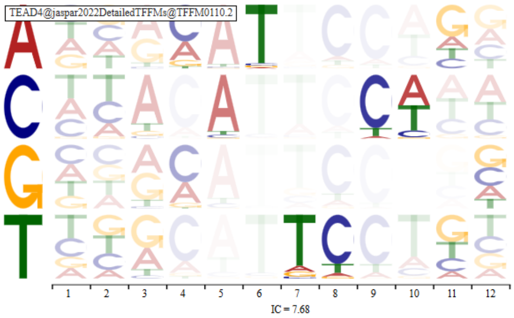

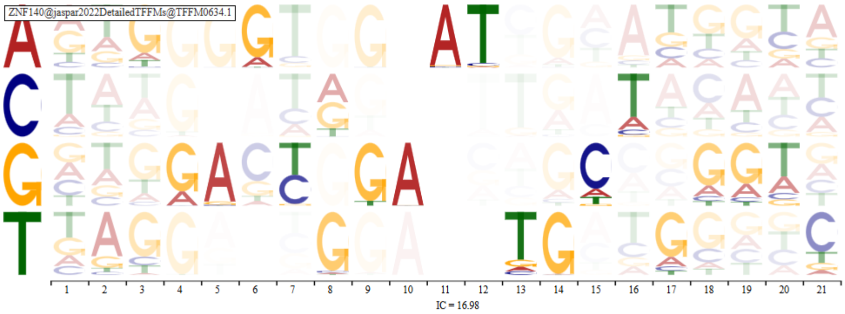

Figure S2: Predicted TFBS loss or gain effect results summary from FABIAN-variant of two putative causal variants (**CHR3:10077301** and **CHR3:10081209**).
